# Plastidial starch phosphorylase regulates maltodextrin turnover during starch granule initiation in Arabidopsis leaves

**DOI:** 10.1093/plphys/kiaf216

**Published:** 2025-06-03

**Authors:** Liping Wang, You Wang, Regina Feil, Gregory J MacNeill, John E Lunn, Ian J Tetlow, Michael J Emes

**Affiliations:** Department of Molecular and Cellular Biology, University of Guelph, Guelph, ON N1G 2W1, Canada; Department of Molecular and Cellular Biology, University of Guelph, Guelph, ON N1G 2W1, Canada; Max Planck Institute of Molecular Plant Physiology, Potsdam-Golm 14476, Germany; Department of Molecular and Cellular Biology, University of Guelph, Guelph, ON N1G 2W1, Canada; Max Planck Institute of Molecular Plant Physiology, Potsdam-Golm 14476, Germany; Department of Molecular and Cellular Biology, University of Guelph, Guelph, ON N1G 2W1, Canada; Department of Molecular and Cellular Biology, University of Guelph, Guelph, ON N1G 2W1, Canada

## Abstract

PLASTIDIAL STARCH PHOSPHORYLASE 1 (PHS1) is considered integral to starch synthesis, yet its role in transient starch synthesis in photosynthetic tissues remains unclear, as mutation of PHS1 in Arabidopsis (*Arabidopsis thaliana*) does not affect the metabolic profile of leaves. PHS1 activity is elevated in the starch branching enzyme *sbe2.1 sbe2.2* double mutant, which lacks starch granules but retains intact genes encoding granule initiation proteins, making it an ideal plant material for exploring PHS1 function. We generated a triple mutant, *sbe2.1 sbe2.2 phs1-1*, which showed additional accumulation of soluble maltodextrins, a loss of insoluble linear α-glucans in the leaves, and substantially retarded plant growth, compared to the *sbe2.1 sbe2.2* double mutant. STARCH SYNTHASE 3 (SS3) and SS4 activities increased in the *sbe2.1 sbe2.2 phs1-1* triple mutant relative to the *sbe2.1 sbe2.2* double mutant. Additional loss of SS4 in the *sbe2.1 sbe2.2 phs1-1* background partially reversed phenotypes observed in the triple mutant: maltodextrin content decreased, insoluble α-glucans reappeared, and plant growth improved. Principal component analysis revealed that the metabolite profile of the *sbe2.1 sbe2.2 ss4* and *sbe2.1 sbe2.2 phs1-1 ss4* mutants, particularly the levels of organic acids from the tricarboxylic acid cycle, more closely resembled that of the wild type than that of *sbe2.1 sbe2.2* and *sbe2.1 sbe2.2 phs1-1*. These findings suggest that PHS1 plays a critical role in maltodextrin turnover and carbon regulation in chloroplasts, maintaining a coordinated balance of synthetic and degradative activities. We propose that PHS1 functions as a metabolic buffer, with its role becoming more crucial when starch synthesis pathways are disrupted.

## Teaser

PHS1 is essential for maltodextrin turnover and carbon balance in the model plant Arabidopsis, its importance becoming more pronounced when starch synthesis is disrupted.

## Introduction

Starch is an insoluble and osmotically inert form of carbohydrate storage found in both leaves and storage organs of most plant species. In Arabidopsis, approximately 30% of carbohydrate derived from photosynthesis is stored in chloroplast starch granules during daytime and is degraded to provide a continuous supply of sugars for plant metabolism and growth during the night (Graf et al. 2010; MacNeill et al. 2017). Starch biosynthesis begins with the elongation of a soluble α-glucan chain primer via transfer of the glucosyl moiety from the soluble precursor ADP-glucose (ADPGlc) by starch synthases (SSs). Subsequently, starch branching enzymes (SBEs) act on the α-1,4 glucosidic bonds to establish α-1,6 glucosidic branches, while debranching enzymes (DBEs) remove excessive branches, facilitating polymer crystallization (Myers et al. 2000; Ball and Morell 2003). Overall, it is a process of glucose (Glc) polymerization involving the conversion of soluble polyglucan/maltodextrin to insoluble starch granules. The process of de novo elongation of soluble α-glucan chains (maltodextrin) is an important process for starch initiation (Malinova et al. 2018). STARCH SYNTHASE 4 (SS4) plays a specific role in generating α-glucan primers for granule initiation and acts upstream of the other SSs (SS1, SS2, and SS3). Loss of *ss4* in Arabidopsis (*Arabidopsis thaliana*) markedly decreases the granule number in chloroplasts and is accompanied by substantial accumulation of ADPGlc (Roldan et al. 2007; Szydlowski et al. 2009; Crumpton-Taylor et al. 2013; Ragel et al. 2013). However, SS4 does not work independently and requires other proteins for assembly of the initiation complex (Malinova et al. 2018). PROTEIN TARGETING TO STARCH 2 (PTST2) was shown to be involved in the starch initiation process by interacting with SS4 and by delivering maltodextrin substrates to SS4 (Seung et al. 2017). In addition, 2 more noncatalytic plastidial proteins, MAR-BINDING FILAMENT-LIKE PROTEIN 1 and PROTEIN INVOLVED IN STARCH INITIATION 1 (PII1) (also known as MYOSIN-RESEMBLING CHLOROPLAST PROTEIN), have been shown to interact with PTST2 (Seung et al. 2018 ; Vandromme et al. 2019). PII1 also interacts with SS4, and simultaneous elimination of PII1 and SS4 in Arabidopsis further reduces starch granule number compared to the *ss4* single mutant (Vandromme et al. 2019 & 2023).

In addition to the SS4-centered protein complex, PLASTIDIAL STARCH PHOSPHORYLASE 1 (PHS1) is another enzyme that can elongate or degrade maltodextrins in order to provide α-glucan primers during the starch initiation process. PHS1, localized in chloroplasts, catalyzes a reversible reaction: α-glucan _(n)_ + glucose-1-phosphate (Glc1P) ⇄ α-glucan _(n_  _+_  _1)_ + inorganic phosphate (Pi). In contrast to the cytosolic isoform termed PHS2, PHS1 has a high affinity for small linear maltodextrins and low affinity for highly branched glucans (Zeeman et al. 2004; Dauvillee et al. 2006). The phosphorolytic activity of maize endosperm PHS1 is favored in the presence of maltooligosaccharides (Mu et al. 2001). Hence, in principle, PHS1 could be involved in maltodextrin metabolism and starch synthesis. Despite catalyzing a reversible reaction, PHS1 was initially thought to favor degradation of α-glucans to provide hexose phosphate based on the low ratio of Glc1P to Pi observed in plant cells (Stitt et al. 1978; Kruger and ap Rees 1983) and there is evidence that phosphorolysis works concurrently with amylolytic activity during nocturnal leaf starch degradation (Weise et al. 2006). However, a study by Hwang et al. (2010) suggested that the catalytic action of PHS1 is not controlled exclusively by the relative concentrations of the substrates Glc1P and Pi. Direct evidence for a potential role in starch biosynthesis was provided showing that PHS1 was involved in incorporation of radiolabeled ^14^C-Glc1P into starch in potato tubers (Fettke et al. 2010 & 2012). Recent studies further demonstrated that in potato tubers, PHS1 was able to produce longer chain-length maltodextrin in plastids with increased availability of Glc1P (Flores-Castellanos and Fettke 2023). The generation of linear long-chain α-glucans by PHS1 was also observed in rice endosperm where its involvement in starch synthesis during seed development was implicated at early stages of α-glucan biosynthesis, as the loss of PHS1 produced smaller starch granules (Satoh et al. 2008). In both potato and rice, PHS1 appeared to be critical for maintaining starch content under low temperature conditions, while PHS1 elimination did not have a substantial impact on starch synthesis under high temperatures, suggesting that other activities compensate for the deficiency of PHS1 under high temperature conditions (Satoh et al. 2008; Fettke et al. 2012; Orawetz et al. 2016). In addition, the plastidic isoform PHS1 in wheat endosperm was recently reported to be involved exclusively in B-type granule initiation through interaction with the putative ortholog of PTST2, B-GRANULE CONTENT 1 (Kamble et al. 2023).

The majority of studies on PHS1 have focused on synthesis of starch in storage tissues, whereas the function of PHS1 in photosynthetic tissues, during transient starch synthesis, remains rather enigmatic. Lack of PHS1 in Arabidopsis does not significantly alter starch content, starch granule number, or plant growth (Zeeman et al. 2004). When introducing the *phs1* mutation into genetic backgrounds with deficiencies in maltose metabolism, such as *dpe2* (lacking DISPROPORTIONATING ENZYME 2, DPE2) or *mex1* (lacking the maltose transporter, MALTOSE EXCESS PROTEIN 1, MEX1), the respective double mutants display additionally retarded growth and less leaf starch under a light–dark regime, compared to the respective *dpe2* and *mex1* single mutants (Malinova et al. 2014 ), implying a key role for PHS1 in starch turnover in Arabidopsis. These results also suggest that the function of PHS1 in Arabidopsis only becomes apparent when starch turnover is compromised. The Arabidopsis *sbe2.1 sbe2.2* double mutant (also known as *be2 be3*; Dumez et al. 2006) lacks both functional SBEs and fails to produce starch granules while accumulating large amounts of maltose (Dumez et al. 2006; Liu et al. 2016). Total starch/α-glucan phosphorylase activities (derived from both PHS1 and PHS2) in leaves of the *sbe2.1 sbe2.2* double mutant are elevated approximately 4-fold compared to that observed in wild-type (WT) leaves (Dumez et al. 2006). The *sbe2.1 sbe2.2* double mutant employed in this study retains intact genes encoding granule initiation proteins and thus provides a unique genetic background to explore why and how PHS activities become elevated. In this study, we demonstrate that both PHS1 and PHS2 activities increase in the *sbe2.1 sbe2.2* double mutant. In order to focus on the starch initiation process in chloroplasts, we generated a homozygous triple mutant between *sbe2.1 sbe2.2* and the *phs1* lines and demonstrate that PHS1 is indispensable for controlling maltodextrin levels as well as maintaining plant growth. Additional mutation of SS4 partially recovered the phenotype of the *sbe2.1 sbe2.2 phs1* triple mutant and was accompanied by a decrease in the amount of maltodextrin accumulated. Collectively, this study provides evidence that PHS1 regulates maltodextrin levels to maintain normal physiological functions in *Arabidopsis* leaf cells.

## Results

### Mutation of *phs1* in the *sbe2.1 sbe2.2* background further reduces plant size

**Figure 1.**
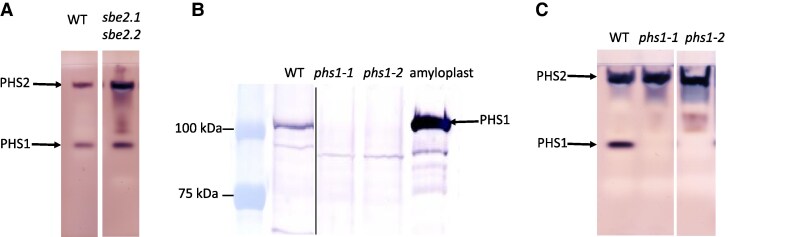
Characterization of starch phosphorylase activities in *sbe2.1 sbe2.2* and *phs1* mutants. Soluble proteins were extracted from 4-wk-old rosette leaves at the end of 16-h light, and extracts containing 100 *µ*g proteins were loaded per lane. **A)** Zymogram of PHS1 (plastidial) and PHS2 (cytosolic) activities in WT and *sbe2.1 sbe2.2* double mutant. **B)** Western blots following SDS-PAGE gel electrophoresis of proteins extracted from the *phs1-1* and *phs1-2* mutants along with WT plant probed with maize anti-PHS1 antibody. A maize endosperm amyloplast extract (5 *µ*g/µL) was used as a positive control. The molecular weights of protein standards are indicated. **C)** Zymogram of PHS1 and PHS2 activities in the *phs1-1* and *phs1-2* mutants along with WT plant. The arrows indicate the plastidial (PHS1) and the cytosolic (PHS2) isoforms.

It was previously reported that in the *sbe2.1 sbe2.2* mutant, starch phosphorylase exhibited phosphorolytic activity 4 times higher than in WT leaves (Dumez et al. 2006). We assessed the catalytic activity (synthetic reaction) of PHS1 on zymograms which have the advantage of separating distinct starch phosphorylase isoforms. Activities of both the plastidic PHS1 and cytosolic PHS2 were increased in the *sbe2.1 sbe2.2* mutant compared to WT, in 25-d-old rosette leaves (Fig. 1A).

**Figure 2.**
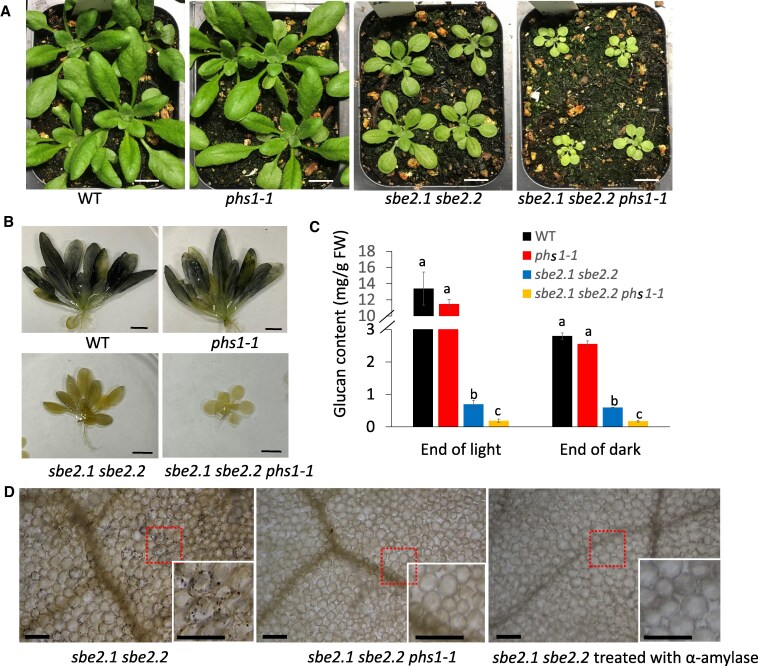
*phs1-1* mutation in *sbe2.1 sbe2.2* background further inhibits plant growth and impedes generation of insoluble α-glucans. **A)** Growth phenotypes of *phs1-1* mutant, *sbe2.1 sbe2.2* double mutant, *sbe2.1 sbe2.2 phs1-1* triple mutant, and the WT plant. Images were captured of plants at 21 d after germination. Scale bars = 1 cm. **B)** Visual assessment of water-insoluble α-glucans in plants. Plants were harvested at the end of the 16-h light period, decolorized, and stained with Lugol's solution. Scale bars = 1 cm. **C)** Insoluble α-glucan contents in leaves collected at the end of the 16-h light period and the end of the 8-h dark period, respectively. All values are means ± SE (*n* = 4, each replicate consisted of a mixture of 5 plants). Different letters within each time point indicate significant differences based on 1-way ANOVA analysis with Tukey's post hoc test (*P* < 0.05). The columns labeled by different letters are significantly different, and those labeled by the same letters are not significantly different. Note the broken *y* axis scale. **D)** Microscope images of leaf mesophyll cells from fully expanded leaves showing insoluble α-glucans in the double mutant (left panel), the absence of insoluble α-glucans in the triple mutant (middle panel), and the removal of insoluble α-glucans by α-amylase in the double mutant (right panel). Each image includes an inset that shows a magnified view of a small portion of the original image (indicated by a red-dotted box). Scale bars = 100 *µ*m.

We generated combined loss-of-function mutants for the plastidial PHS1 and SBEs to investigate their functions in starch metabolism within the chloroplasts. First, we identified 2 independent homozygous T-DNA insertion knockout mutants, *phs1-1* (SALK_055562) and *phs1-2* (GK-257A06) using 2 gene-specific primers and the T-DNA left border primers. The T-DNA insertion sites in *phs1-1* and *phs1-2* were located in the fourth and second exons of *PHS1*, respectively (Supplementary Fig. S1, A and B). The lack of detectable PHS1 protein and catalytic activity confirmed both mutants to be null alleles (Fig. 1, B and C). Two triple mutants were generated by crossing the *sbe2.1 sbe2.2* double mutant with either *phs1-1* or *phs1-2*. Under normal 16-h light/8-h dark growth conditions, the *sbe2.1 sbe2.2* double mutant displayed a lower growth rate and reduced plant size (Dumez et al. 2006; Fig. 2A), whereas no discernible morphological differences were observed in the single mutants, *phs1-1* or *phs1-2* (Fig. 2A; Supplementary Fig. S2A). Both triple mutants, *sbe2.1 sbe2.2 phs1-1* and *sbe2.1 sbe2.2 phs1-2*, were fertile and able to produce seeds, but plants were notably smaller from an early seedling stage, compared to the *sbe2.1 sbe2.2* double mutant (Fig. 2A; Supplementary Fig. S2A). Fresh weight (FW) of above-ground tissues of the *sbe2.1 sbe2.2 phs1-1* triple mutant was approximately two-thirds of that in the *sbe2.1 sbe2.2* mutant at 10 d after germination (Supplementary Fig. S2B). By 25 d after germination, the FW of the *sbe2.1 sbe2.2 phs1-1* mutant was only one-third of the *sbe2.1 sbe2.2* double mutant (Supplementary Fig. S2B), confirming the slower growth in the triple mutant. The 2 triple mutants *sbe2.1 sbe2.2 phs1-1* and *sbe2.1 sbe2.2 phs1-2* exhibited similar phenotypes, suggesting that the additional decrease in plant size in the 2 triple mutants was caused by PHS1 deficiency. In subsequent experiments, we used the *sbe2.1 sbe2.2 phs1-1* mutant.

### Mutation of *phs1* in the *sbe2.1 sbe2.2* background impedes generation of insoluble α-glucans

The *sbe2.1 sbe2.2* double mutant is not able to produce normal starch granules due to absence of SBE activity (Dumez et al. 2006). Nevertheless, leaves of *sbe2.1 sbe2.2* plants were still lightly stained when treated with iodine solution, whereas *sbe2.1 sbe2.2 phs1-1* leaves were not (Fig. 2B). We then measured the contents of starch/insoluble α-glucans in leaves, collected at 25 d after germination, at the end of the light and dark periods (16-h day/8-h night), respectively. Starch contents in the leaves of the single mutant *phs1-1* were not significantly different from WT (11.5 ± 0.6 mg/g FW at end of light; 2.6 ± 0.1 mg/g FW at the end of dark) (Fig. 2C). In the *sbe2.1 sbe2.2* mutant, much lower but detectable amounts of insoluble α-glucans were consistently observed at the end of light (0.69 ± 0.12 mg/g FW) and end of dark (0.59 ± 0.02 mg/g FW) in plants grown from multiple batches (Fig. 2C). Distinct from the α-glucans/starch in WT and *phs1-1*, the insoluble α-glucans in *sbe2.1 sbe2.2* did not undergo any evident diurnal turnover (Fig. 2C). When compared to the *sbe2.1 sbe2.2* double mutant, the insoluble α-glucans in the leaves of *sbe2.1 sbe2.2 phs1-1* triple mutant were even lower at the end of light (0.18 ± 0.06 mg/g FW) and end of dark (0.17 ± 0.03 mg/g FW) and at the limit of detection.

To explore the properties and location of these α-glucans, *sbe2.1 sbe2.2* and *sbe2.1 sbe2.2 phs1-1* ethanol-cleared leaves were stained with iodine solution and closely examined under the microscope. In fully expanded rosette leaves, numerous dark spots were observed in the mesophyll cells of the *sbe2.1 sbe2.2* mutant (Fig. 2D, left panel), while no such structures were identifiable in *sbe2.1 sbe2.2 phs1-1* leaves (Fig. 2D, middle panel). We applied α-amylase to the ethanol-cleared leaves from the *sbe2.1 sbe2.2* double mutant to see whether these structures can be digested amylolytically. After incubating at 37 °C for 4 h, all the iodine-stained structures disappeared (Fig. 2D, right panel), consistent with the premise that they are linear α-glucans.

**Figure 3.**
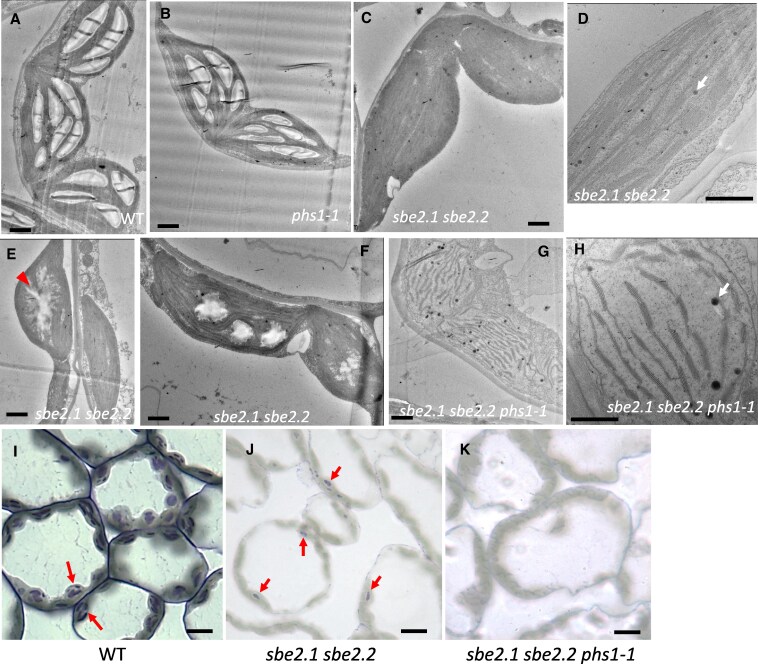
TEM and light microscope analyses of leaf sections. Four-week-old rosette leaves were collected at the end of the 16-h light period for imaging. **A** to **H)** TEM images: **A)** WT; **B)**  *phs1-1* mutant; and **C** to **F)**  *sbe2.1 sbe2.2* double mutant. Panel **C)** displays representative chloroplasts lacking granule-like structures, while Panel **D)** provides a magnified view of thylakoid membranes in the double mutant. Panels **E)** and **F)** show chloroplasts containing various morphologies of irregular granule-like structures. Arrowhead in **E)** indicates diffused boundary of the granule-like structure. Panels **G)** and **H)** depict the *sbe2.1 sbe2.2 phs1-1* triple mutant, with **G)** showing poorly defined chloroplast membrane boundaries and **H)** revealing fewer assembled thylakoid membranes and expanded stroma space. Arrows in **D)** and **H)** indicate plastoglobules. Scale bars = 1 *µ*m. Panels **I)** to **K)** show light microscope images: Semi-thin sections were stained with toluidine blue for 5 min. Arrows indicate starch granules in WT (I) or granule-like structures in *sbe2.1 sbe2.2*  **J)**. No granule-like structures were observed in *sbe2.1 sbe2.2 phs1-1*  **K)**. Scale bars = 10 *µ*m.

The ultrastructure of the chloroplasts was examined using transmission electron microscopy (TEM). Mature leaf tissues were harvested at 25 d after germination, at the end of the 16-h light period. Both WT and *phs1-1* chloroplasts typically contained around 4 to 7 ellipsoid starch granules (Fig. 3, A and B). The *sbe2.1 sbe2.2* chloroplasts were free of normal starch granules as expected. Based on the observation of 140 chloroplasts in the *sbe2.1 sbe2.2* mutant, approximately 80% (110 out of 140) contained only thylakoids and stroma (Fig. 3C), whereas 20% (30 out of 140) contained starch granule-like structures which were of irregular shape and of various sizes, ranging from 1 to 4 *μ*m in length, compared with relatively smooth and uniform structures in WT. Some of these granular structures were disorganized, the boundaries of which appeared more diffuse (Fig. 3E), while some were more rounded but with rough boundaries (Fig. 3F). By contrast, no such structures were found after examining 100 chloroplasts in the *sbe2.1 sbe2.2 phs1-1* triple mutant. Instead, the majority of chloroplasts in the triple mutant were misshapen and swollen, compared to WT and the *sbe2.1 sbe2.2* double mutant (Fig. 3, A and C to F), and membrane boundaries of adjacent chloroplasts were undefined (Fig. 3G). In the *sbe2.1 sbe2.2* double mutant, the thylakoid grana comprised an average of 10.2 ± 1.7 stacks (*n* = 20, mean ± SD; Fig. 3D), while in the *sbe2.1 sbe2.2 phs1-1* triple mutant, only 3.7 ± 0.5 thylakoid stacks (*n* = 20, mean ± SD) were appressed together, with larger stromal spaces between granal stacks (Fig. 3H). In addition, in both double and triple mutants, more plastoglobules were observed in the chloroplasts (Fig. 3, D and H).

The starch granule-like structures observed in approximately 20% of the chloroplasts of the *sbe2.1 sbe2.2* mutant may account for the dark spots seen in leaves stained with iodine solution. To further investigate this, we examined semi-thin sections of rosette leaves, followed by toluidine blue staining, which has been used to detect starch granules under the light microscope (Crumpton-Taylor et al. 2013; Lu et al. 2018). Consistent with the observations from electron micrographs, multiple starch granules were observed in each WT chloroplast (Fig. 3I), while in *sbe2.1 sbe2.2*, of approximately 10 chloroplasts per cell observed in the evaluated cross sections, only 1 to 3 chloroplasts contained stained starch granule-like structures (Fig. 3J). As expected, no stained structures were observed in the chloroplasts of the triple mutant (Fig. 3K). The above results demonstrate that additional deficiency of *phs1* in the *sbe2.1 sbe2.2* background impedes the generation of insoluble linear α-glucans and impacts chloroplast development.

### Mutation of *phs1* in the *sbe2.1 sbe2.2* background results in additional accumulation of soluble α-glucans

Leaves of *sbe2.1 sbe2.2* plants accumulated high contents of soluble α-glucans, approximately 80% of which was maltose (Dumez et al. 2006). To explore the impacts of the *phs1* mutation, the contents of soluble sugars/α-glucans were measured in rosette leaves of all lines. The results show that the Glc contents in *sbe2.1 sbe2.2* and *sbe2.1 sbe2.2 phs1-1* were higher than in either WT or the *phs1-1* single mutant, at the end of both the light and dark periods (Table 1). The most dramatic differences observed were in the contents of soluble α-glucans including maltose and maltodextrin. The maltose level in *sbe2.1 sbe2.2* was 40-fold higher than in WT and the *phs1-1* single mutant and was further elevated in *sbe2.1 sbe2.2 phs1-1* plants which contained an additional 50% and 100% higher maltose, at the end of the light period and end of the dark period, respectively, compared to *sbe2.1 sbe2.2* (Table 1). Maltodextrins accounted for 10% of the total soluble α-glucans and were more than 45 times higher in *sbe2.1 sbe2.2* than in either WT or the *phs1-1* mutant at the end of day and end of night. In the *sbe2.1 sbe2.2 phs1-1* triple mutant, maltodextrin content was 2.5-fold higher than that of the *sbe2.1 sbe2.2* mutant both at the end of light period and end of dark period (Table 1). The observation that *phs1* deficiency significantly increased maltose and maltodextrin amounts in the *sbe2.1 sbe2.2* genetic background suggests that PHS1 plays a role in soluble α-glucan catabolism.

**Table 1.** Soluble sugars and α-glucans (mg/g FW)

Time point	Genotype	Glucose	Fructose	Sucrose	Maltose	Maltodextrin
End of the light	WT	0.58 ± 0.01^a^	0.18 ± 0.02^a^	0.49 ± 0.02^a^,^b^	0.19 ± 0.01^a^	0.02 ± 0.01^a^
	*phs1-1*	0.56 ± 0.03^a^	0.19 ± 0.01^a^	0.46 ± 0.01^a^	0.20 ± 0.01^a^	0.02 ± 0.01^a^
	*sbe2.1 sbe2.2*	1.76 ± 0.08^b^	0.25 ± 0.03^b^	0.52 ± 0.01^b^	7.71 ± 0.10^b^	0.91 ± 0.09^b^
	*sbe2.1 sbe2.2 phs1-1*	1.53 ± 0.04^c^	0.19 ± 0.01^a^	0.59 ± 0.01^c^	11.32 ± 0.23^c^	2.33 ± 0.08^c^
End of the dark	WT	0.61 ± 0.04^a^	0.17 ± 0.01^a^	0.30 ± 0.03^a^	0.18 ± 0.03^a^	0.02 ± 0.01^a^
	*phs1-1*	0.72 ± 0.10^a^	0.17 ± 0.01^a^	0.26 ± 0.04^a^	0.20 ± 0.03^a^	0.02 ± 0.01^a^
	*sbe2.1 sbe2.2*	0.97 ± 0.05^b^	0.26 ± 0.02^b^	0.22 ± 0.04^a^	5.93 ± 0.37^b^	1.15 ± 0.03^b^
	*sbe2.1 sbe2.2 phs1-1*	0.93 ± 0.11^b^	0.19 ± 0.02^a^	0.27 ± 0.01^a^	11.76 ± 0.39^c^	2.76 ± 0.12^c^

Plants were grown under 16-h light/8-h dark conditions and rosette leaves were harvested at 28 d after germination. All values are means ± SE (3 replicates from a mixture of 10 plants). Different letters (a,b,c) within each column indicate significant differences across different genotypes based on 1-way ANOVA analysis with Tukey's post hoc test (*P* < 0.05).

^abc^Different letters (a, b, c) within each column indicate significant differences across different genotypes based on 1-way ANOVA analysis with Tukey's post hoc test (*P* < 0.05).

### SS activity is elevated in the *sbe2.1 sbe2.2 phs1-1* triple mutant

**Figure 4.**
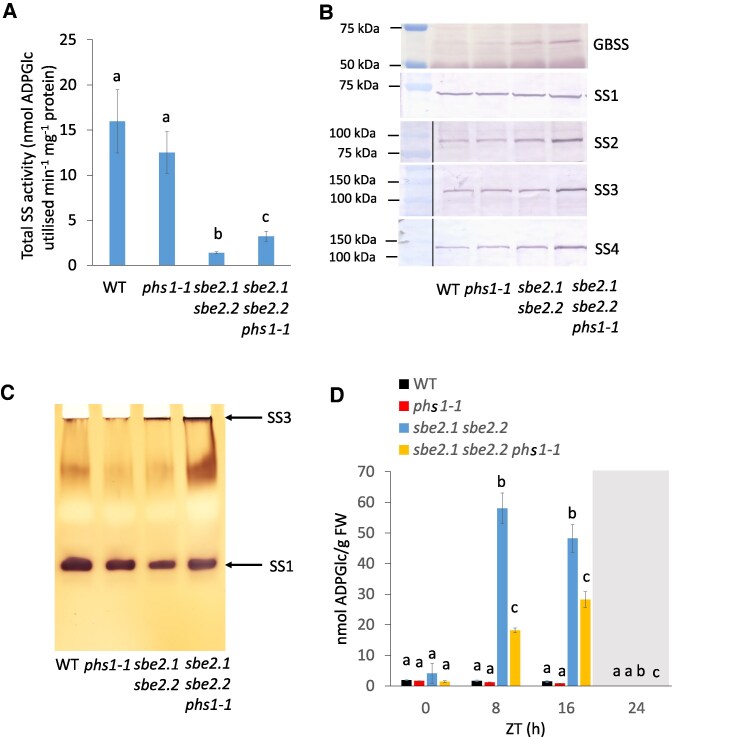
The *sbe2.1 sbe2.2 phs1-1* triple mutant possesses higher SS activity and isoform content compared to the *sbe2.1 sbe2.2* double mutant. **A)** Total soluble SS activity measured from the 4-wk-old rosette leaves collected at the end of the 16-h light period. Values represent means ± SE (*n* = 3, leaves were pooled from at least 10 plants). Different letters indicate significant differences based on 1-way ANOVA analysis with Tukey's post hoc test (*P* < 0.05). **B)** Comparison of SS1, SS2, SS3, SS4, and GBSS protein levels via western blot, using the corresponding peptide-specific antibodies. The molecular weights of protein standards are indicated. **C)** Zymogram showing SS1 and SS3 (and SS4) activity bands, identified using specific antibodies. **D)** ADPGlc content determined by LC-MS/MS. Four-week-old rosette leaves were harvested at 0, 8, 16, and 24 h during a diurnal cycle (16-h light/8-h dark). The shaded area indicates the 8-h dark phase (16 to 24 h). ZT represents Zeitgeber time (i.e. time since the previous dawn). Values are means ± SE (*n* = 4 or 5, leaves were pooled from at least 10 plants). Different letters within each time point indicate significant differences across different genotypes based on 1-way ANOVA analysis with Tukey's post hoc test (*P* < 0.05).

Since the *phs1* mutation interrupted both insoluble and soluble α-glucan metabolism in the *sbe2.1 sbe2.2* mutant background, we next conducted a series of assays of other enzymes involved in starch metabolism. Enzymatic assays of ADPGlc pyrophosphorylase (AGPase), SSs, disproportionating enzymes (DPEs), and β-amylase were conducted. Total soluble SS activity was 3 times higher in the *sbe2.1 sbe2.2 phs1-1* triple mutant compared to the *sbe2.1 sbe2.2* double mutant, but considerably lower than that of WT and *phs1-1* (Fig. 4A). Other enzymes showed comparable activities between *sbe2.1 sbe2.2* and *sbe2.1 sbe2.2 phs1-1* (Supplementary Fig. S3). Since there are 5 SS isoforms and 1 granule bound SS (GBSS) in the Arabidopsis genome, we first examined their transcript levels by conducting RT-PCR using vegetative leaves collected at the end of 16-h light period. The results showed no significant changes between *sbe2.1 sbe2.2* and *sbe2.1 sbe2.2 phs1-1* in the expression of any of these genes (Supplementary Fig. S4). Even so, western blot analysis revealed that GBSS, SS2, SS3, and SS4, but not SS1, proteins were enriched in *sbe2.1 sbe2.2 phs1-1*, compared to *sbe2.1 sbe2.2* (Figure 4B). Zymogram analysis was employed to examine activities of individual SS isoforms. Previous studies in Arabidopsis indicate that the very top band(s) of SS zymograms represents the activity of SS3, while the lowest migrating band indicates SS1 activity (Szydlowski et al. 2009, Fig. 4C). SS3 activity in the *sbe2.1 sbe2.2 phs1-1* triple mutant was markedly stronger than that in *sbe2.1 sbe2.2* (Figure 4C), implying that the absence of PHS1 corresponded with increased SS3 activity in *sbe2.1 sbe2.2 phs1-1*. ADPGlc is the soluble substrate for SSs, and the *sbe2.1 sbe2.2* double mutant contained the highest levels of this metabolite, which was more than 30 times that of WT during the light period measured at 8 and 16 h (Fig. 4D). The *sbe2.1 sbe2.2 phs1-1* triple mutant showed only 30% and 60% of the ADPGlc content found in the *sbe2.1 sbe2.2* double mutant after 8 and 16 h light, respectively (Fig. 4D), consistent with the increase in total SS activity observed and increased SS2, SS3, and SS4 protein levels in the *sbe2.1 sbe2.2 phs1-1* triple mutant (Fig. 4, A and C). Despite differences in amounts of ADPGlc, both the *sbe2.1 sbe2.2* double and *sbe2.1 sbe2.2 phs1-1* triple mutants shared a similar pattern of ADPGlc turnover during a diurnal cycle (Fig. 4D). There are only trace amounts of ADPGlc in all the lines at night (Fig. 4D), consistent with there being little or no ADPGlc synthesis in the dark partially due to redox inactivation of AGPase (Hendriks et al. 2003; Hädrich et al. 2012).

### Mutation of *ss4* in the *sbe2.1 sbe2.2 phs1-1* background improves plant growth and reverses the formation of insoluble linear α-glucans

**Figure 5.**
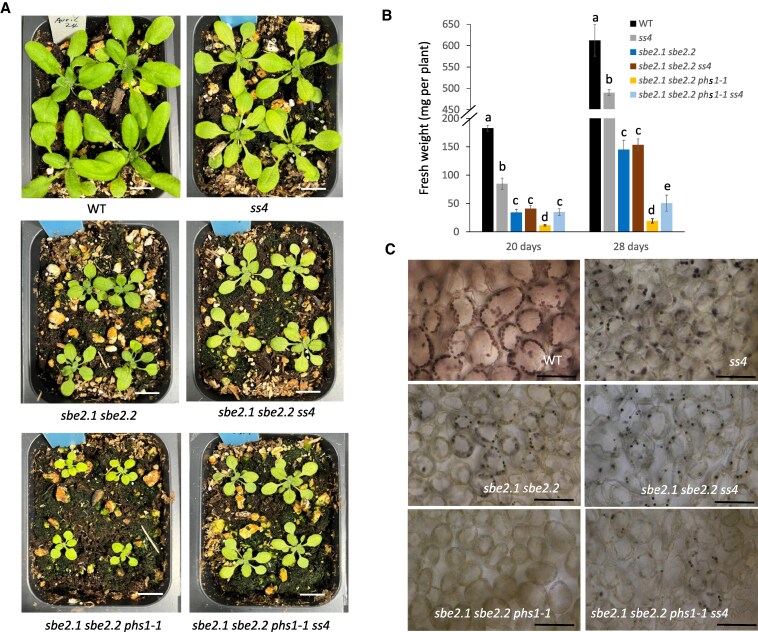
The *sbe2.1 sbe2.2 phs1-1* triple mutant phenotype is partially rescued by the additional loss of *SS4*. **A)** Growth phenotypes across different mutant combinations. Images were captured of plants at 20 d postgermination. Scale bars = 1 cm. **B)** FW of the above-ground tissues of WT plants and mutant lines at 20 and 28 d of age. Values are means ± SE (*n* = 15 to 20). Different letters within each time point indicate significant differences based on 1-way ANOVA analysis with Tukey's post hoc test (*P* < 0.05). Note the broken *y* axis scale. **C)** Light microscope images of leaf mesophyll cells showing reappearance of insoluble α-glucans in the *sbe2.1 sbe2.2 phs1-1 ss4* mutant. Leaves were harvested at the end of the 16-h light period. Scale bars = 200 *µ*m.

SS4 is thought to provide the α-glucan primer for the actions of other SSs (Crumpton-Taylor et al. 2013; Ragel et al. 2013) and is essential for the starch initiation process in Arabidopsis (Lu et al. 2018). Given that SS4 is more active in the *sbe2.1 sbe2.2 phs1-1* triple mutant than in the *sbe2.1 sbe2.2* double mutant (Fig. 4B), we next introduced an *ss4* mutation into the *sbe2.1 sbe2.2 phs1-1* background to assess whether the additional loss of SS4 would affect plant growth and carbohydrate metabolism. Crosses between *sbe2.1 sbe2.2 phs1-1* and an *ss4* single mutant (CS2103513) as well as a cross of *sbe2.1 sbe2.2* with the *ss4* mutant were conducted to generate F_2_ populations and were used to identify homozygous mutants *sbe2.1 sbe2.2 phs1-1 ss4* and *sbe2.1 sbe2.2 ss4*, respectively. These genetically characterized mutant lines were grown under 16-h light/8-h dark diel cycles together with the previously described single, double, and triple mutants. The *ss4* single mutant was slightly smaller than WT, as reported previously (Roldan et al. 2007; Crumpton-Taylor et al. 2013; Ragel et al. 2013; Malinova et al. 2014, Fig. 5, A and B). Combining the *ss4* mutation with *sbe2.1 sbe2.2 phs1-1* might be expected to further impact plant growth. However, the *sbe2.1 sbe2.2 phs1-1 ss4* mutant had an obviously larger leaf shoot size than that of the *sbe2.1 sbe2.2 phs1-1* triple mutant (Fig. 5A). On the basis of above ground FW, *sbe2.1 sbe2.2 phs1-1 ss4* plants were approximately 3 times as large as *sbe2.1 sbe2.2 phs1-1* when measured after 20 d (11.3 mg vs. 34.8 mg) and 28 d (19.2 mg vs. 50.6 mg) postgermination (Fig. 5B). However, *sbe2.1 sbe2.2 ss4* did not exhibit significant differences in shoot size and FW when compared to *sbe2.1 sbe2.2* plants (Fig. 5, A and B).

**Figure 6.**
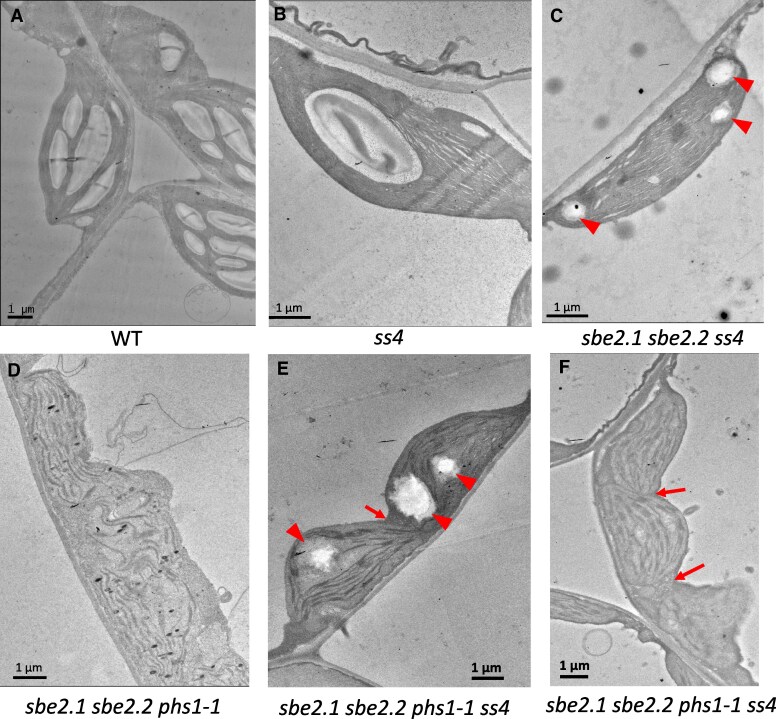
TEM ultrastructure of the *sbe2.1 sbe2.2 phs1-1 ss4* mutant. **A)** WT; **B)**  *ss4* single mutant; **C)**  *sbe2.1 sbe2.2 ss4* mutant (granule-like structures indicated by arrowheads); **D)**  *sbe2.1 sbe2.2 phs1-1* triple mutant; **E** and **F)**  *sbe2.1 sbe2.2 phs1-1 ss4* mutant. Approximately one-quarter of the chloroplasts containing one or more granule-like structures in (E), indicating the formation of insoluble linear α-glucans (arrowheads). Panel **F)** indicates chloroplasts with no granule-like structures. Arrows in **E)** and **F)** indicate distinct chloroplast envelope membrane boundaries. Leaves were harvested at the end of the 16-h light period. Scale bars = 1 *µ*m.

We had shown that the iodine-stained structures in *sbe2.1 sbe2.2*, representing insoluble linear α-glucans, disappeared in the *sbe2.1 sbe2.2 phs1-1* triple mutant (Fig. 2D). However, these structures reappeared in the leaves of the *sbe2.1 sbe2.2 phs1-1 ss4* mutant at the end of 16 h light period when stained with iodine solution (Fig. 5C). We then examined the ultrastructure of these leaf cells using TEM. Consistent with a previous report (Malinova et al. 2017), most chloroplasts in the *ss4* single mutant contained a single starch granule (Fig. 6B). Similar to *sbe2.1 sbe2.2*, approximately 25% (34 out of 134) of the chloroplasts in *sbe2.1 sbe2.2 ss4* leaves contained starch granule-like structures (Fig. 6C, arrowheads). In contrast to the *sbe2.1 sbe2.2 phs1-1* triple mutant, where no such structures were observed (Fig. 6D), the introduction of the *ss4* mutation into this genetic background led to the formation of starch granule-like structures in a similar proportion (31 out of 131 chloroplasts) (Fig. 6E, arrowheads). Further, the outer envelope of chloroplasts in the *sbe2.1 sbe2.2 phs1-1* mutant was ill defined compared to WT (Fig. 6D). In contrast, the boundaries of the outer envelope in the *sbe2.1 sbe2.2 phs1-1 ss4* mutant were much more apparent (Fig. 6, E and F, arrows).

We further quantified the contents of insoluble α-glucans in 28-d-old leaves collected at the end of the 16-h light period and end of the 8-h dark period. Compared to WT, *ss4* leaves accumulated only half the starch content at the end of the light period, but contained a comparable level of starch at the end of the dark period (Table 2). Consistent with the microscopy observations (Fig. 6), the insoluble α-glucan content in *sbe2.1 sbe2.2 phs1-1 ss4* was nearly 4 times greater than in the *sbe2.1 sbe2.2 phs1-1* triple mutant at the end of the light and dark periods (Table 2). Similarly, the content in *sbe2.1 sbe2.2 ss4* was doubled, both at the end of the light and dark periods, when compared with the *sbe2.1 sbe2.2* leaves. However, the insoluble α-glucan content in the *sbe2.1 sbe2.2 phs1-1 ss4* mutant was only half of that in *sbe2.1 sbe2.2 ss4* both at the end of the light and dark periods. Although these insoluble α-glucans represent relatively small amounts, the differences with and without SS4 were statistically significant (*P* < 0.05; Table 2) when assessed from multiple batches of plants. In contrast to starch granules in WT or the *ss4* single mutant, the diurnal turnover of these linear α-glucans was negligible (Table 2).

**Table 2.** Starch/insoluble polyglucans and soluble α-glucans (mg/g FW)

Genotype	End of the light			End of the dark		
	Starch/glucan	Maltose	Maltodextrin	Starch/glucan	Maltose	Maltodextrin
WT	17.36 ± 2.02^a^	0.18 ± 0.03^a^	0.03 ± 0.02^a^	3.75 ± 0.26^a^	0.15 ± 0.01^a^	0.09 ± 0.01^a^
*ss4*	8.21 ± 0.34^b^	0.05 ± 0.01^b^	0.06 ± 0.01^a^	3.69 ± 0.04^a^	0.06 ± 0.01^b^	0.12 ± 0.01^a^
*sbe2.1 sbe2.2*	0.62 ± 0.03^c^	12.48 ± 0.13^c^	1.21 ± 0.09^b^	0.47 ± 0.01^b^	7.79 ± 0.21^c^	1.54 ± 0.22^b^
*sbe2.1 sbe2.2 ss4*	1.24 ± 0.06^d^	2.21 ± 0.05^d^	0.04 ± 0.01^a^	1.01 ± 0.03^c^	1.13 ± 0.21^d^	0.33 ± 0.12^c^
*sbe2.1 sbe2.2 phs1-1*	0.16 ± 0.02^e^	15.65 ± 0.38^e^	2.98 ± 0.34^c^	0.16 ± 0.02^d^	13.11 ± 0.55^e^	3.93 ± 0.55^d^
*sbe2.1 sbe2.2 phs1-1 ss4*	0.58 ± 0.04^c^	2.24 ± 0.48^d^	0.06 ± 0.02^a^	0.58 ± 0.03^e^	2.95 ± 0.29^f^	0.50 ± 0.02^e^

Plants were grown under 16-h light/8-h dark conditions and harvested at 28 d after germination. All values are means ± SE (3 replicates from a mixture of 10 plants). Different letters (a,b,c,d,e,f) within each column indicate significant differences across different genotypes based on 1-way ANOVA analysis with Tukey's post hoc test (*P* < 0.05).

### Mutation of *ss4* in the *sbe2.1 sbe2.2 phs1-1* background substantially decreases soluble α-glucan contents

In WT and the *ss4* single mutant, both maltose and maltodextrin were present at low levels (<0.2 mg/g FW) in leaves collected at the end of the 16-h light period and the 8-h dark period. However, introgression of the *ss4* mutation into the *sbe2.1 sbe2.2 phs1-1* mutant significantly reduced the maltose and maltodextrin contents at both time points compared to *sbe2.1 sbe2.2* and *sbe2.1 sbe2.2 phs1-1*. At the end of the light period, although the maltose content in *sbe2.1 sbe2.2 phs1-1* was 30% higher than in the double mutant, additional loss of *ss4* in the above triple mutant decreased the maltose content by 85% (from 15.65 ± 0.38 to 2.24 ± 0.48 mg/g FW), bringing it to a level comparable to that of the *sbe2.1 sbe2.2 ss4* mutant (2.21 ± 0.05 mg/g FW). Similarly, in the *sbe2.1 sbe2.2 phs1-1 ss4* mutant, the maltodextrin content was markedly decreased to levels (0.06 ± 0.02 mg/g FW) similar to those observed in WT plants (0.03 ± 0.02 mg/g FW) (Table 2). At the end of the dark period, maltose and maltodextrin contents in the *sbe2.1 sbe2.2 phs1-1 ss4* mutant were significantly decreased compared to the *sbe2.1 sbe2.2 phs1-1* triple mutant but remained higher than in the *sbe2.1 sbe2.2 ss4* mutant (Table 2), suggesting that PHS1 is involved in metabolizing maltodextrins in the dark period. The significant changes in soluble maltodextrins caused by the additional mutation of *ss4* were further confirmed through thin layer chromatography (TLC) analysis, where the maltose samples from the *sbe2.1 sbe2.2 phs1-1 ss4* and *sbe2.1 sbe2.2 ss4* mutants appeared stained with much less intensity compared to the *sbe2.1 sbe2.2 phs1-1* and *sbe2.1 sbe2.2* mutants (Supplementary Fig. S5).

**Figure 7.**
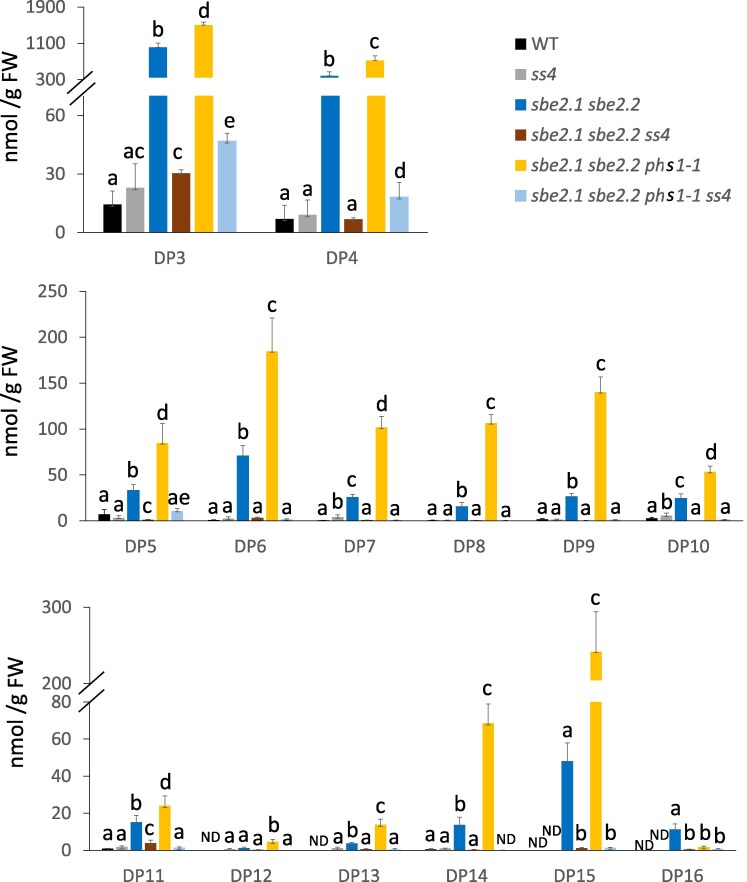
Analyses of maltodextrin patterns in WT and mutant lines. Four-week-old rosette leaves were collected at the end of the 16-h light period. Maltodextrins with degrees of polymerization (DP) 3 to 16 are shown across 3 separate charts. The total soluble sugars used for generating these maltodextrin patterns were the same samples referenced in Table 2. Values are presented as means ± SE (*n* = 3). For a given maltodextrin, different letters indicate significant differences based on 1-way ANOVA analysis with Tukey's post hoc test (*P* < 0.05). ND, not detected. Note the broken *y* axis scale in the upper and bottom charts.

Since the majority of soluble maltodextrins is maltose, it is technically difficult to assess each individual higher-order maltodextrin (DP > 2) by enzymatic methods. We therefore separated the above soluble α-glucan extracts collected at the end of the 16-h light period using Dionex Ion Exchange Chromatography and compared the peaks with a maltodextrin standard. The content of each maltodextrin with different DPs was calculated based on the ratio of peak areas to the total maltodextrin amount determined enzymatically (Fig. 7). Maltotriose (DP3) and maltotetraose (DP4) were the major maltodextrins, other than maltose, which accumulated in the *sbe2.1 sbe2.2* and *sbe2.1 sbe2.2 phs1-1* mutants, with highest levels observed in the latter mutant. Alpha-glucans with a DP > 4 were present at very low levels in the WT and *ss4* mutants. However, in the *sbe2.1 sbe2.2 phs1-1* triple mutant, higher-order maltodextrins of up to DP16 were clearly observed. Similar peaks, though in lower amounts, were also detected in the *sbe2.1 sbe2.2* mutant compared to the *sbe2.1 sbe2.2 phs1-1* mutant. All these α-glucan species were significantly decreased by the additional loss of *ss4* (Fig. 7; Supplementary Fig. S6).

### Mutation of *ss4* in the *sbe2.1 sbe2.2 phs1-1* background reveals changes in other enzyme activities

**Figure 8.**
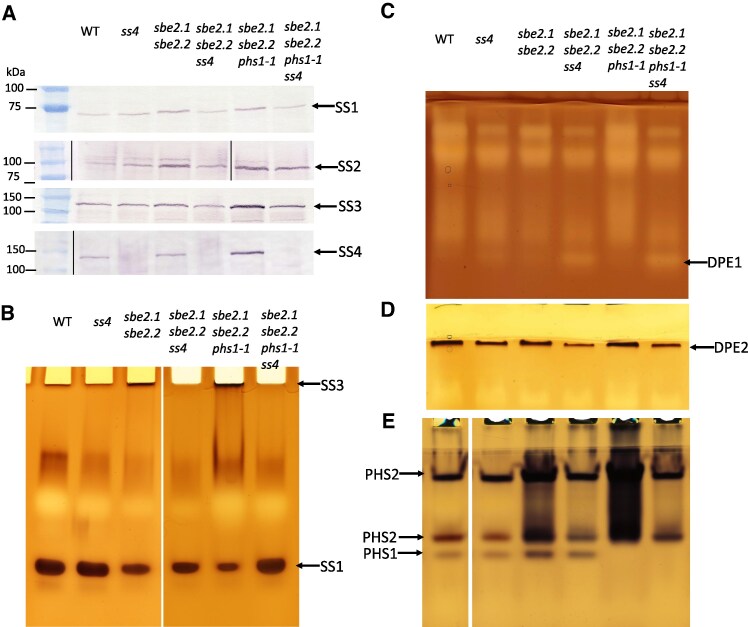
Analysis of protein levels and enzyme activities in SSs, DPEs, and PHSs across different mutant combinations. **A)** Comparison of protein levels of SS1, SS2, and SS3 via western blot using peptide-specific antibodies. Molecular weight standards are indicated. **B)** Zymogram analysis of SS1 and SS3 activities. **C)** Zymogram analysis of DPE1 activity, utilizing maltotriose as the substrate. **D)** Zymogram analysis of DPE2 activity, utilizing maltose as the substrate. **E)** Zymogram analysis of PHS1 and PHS2 activities. Four-week-old rosette leaves were harvested at the end of the 16-h light period.

We examined whether the additional loss of *ss4* affected the other SS isoforms (SS1 to 3). Western blot and zymogram analyses were performed using leaf samples collected at the end of the 16-h light period. Noticeable decreases in SS1, SS2, and SS3 protein levels were observed in the *sbe2.1 sbe2.2 phs1-1 ss4* mutant compared to the *sbe2.1 sbe2.2 phs1-1* triple mutant (Fig. 8A). Similarly, levels of the same proteins were decreased in the *sbe2.1 sbe2.2 ss4* mutant compared to the *sbe2.1 sbe2.2* double mutant (Fig. 8A). Zymogram assays of SS activity indicated that the top bands, representing the SS3 activity, were undetectable in the *sbe2.1 sbe2.2 ss4* and *sbe2.1 sbe2.2 phs1-1 ss4* mutants (Fig. 8B), suggesting the SS3 activity was compromised in these mutants carrying the *ss4* mutation (Fig. 8A). SS1 activity in the *sbe2.1 sbe2.2 phs1-1 ss4* mutant was higher than in the *sbe2.1 sbe2.2 phs1-1* triple mutant (Fig. 8B), although this was not reflected at the protein level (Fig. 8A). Notably, SS1 activity in the *sbe2.1 sbe2.2 phs1-1 ss4* mutant remained comparable to that observed in WT (Fig. 8B). It was unclear which band corresponded to SS2, possibly because the majority of SS2 activity is located in the starch granules.

DPE1 and DPE2 are involved in maltodextrin metabolism in the chloroplast and cytosol, respectively. The DPE1 zymogram was performed as described by Critchley et al. (2001), and the DPE1-specific band was identified by comparison with the *dpe1* mutant (Supplementary Fig. S7). Notably, DPE1 showed higher activity in the *sbe2.1 sbe2.2 ss4* and *sbe2.1 sbe2.2 phs1-1 ss4* mutants compared to the single *ss4* mutant, the *sbe2.1 sbe2.2* double mutant, *sbe2.1 sbe2.2 phs1-1* triple mutant, and WT (Fig. 8C). In contrast, the loss of SS4 in the *sbe2.1 sbe2.2* and *sbe2.1 sbe2.2 phs1-1* mutants appeared to cause a decreased DPE2 activity, relative to the levels in the *sbe2.1 sbe2.2* and *sbe2.1 sbe2.2 phs1-1* mutants (Fig. 8D). We also assessed PHS1 activity. It is worth noting that the loss of SS4 resulted in decreased PHS1 activity in the *sbe2.1 sbe2.2 ss4* mutant compared to the *sbe2.1 sbe2.2* double mutant, though activity remained higher than in WT (Fig. 8E). Given that elimination of the chloroplastic amylase AMY3 in the *ss4* mutant increases the starch granule number and suppresses the pale phenotype (Seung et al. 2016), we also examined α-amylase activity in our mutant lines. Consistent with the findings of Seung et al., we observed a decrease in a major band of α-amylase activity in the lines carrying the *ss4* mutation (Supplementary Fig. S8).

### Mutation of *ss4* in the *sbe2.1 sbe2.2 phs1-1* background partially restores WT-like metabolite profiles

**Figure 9.**
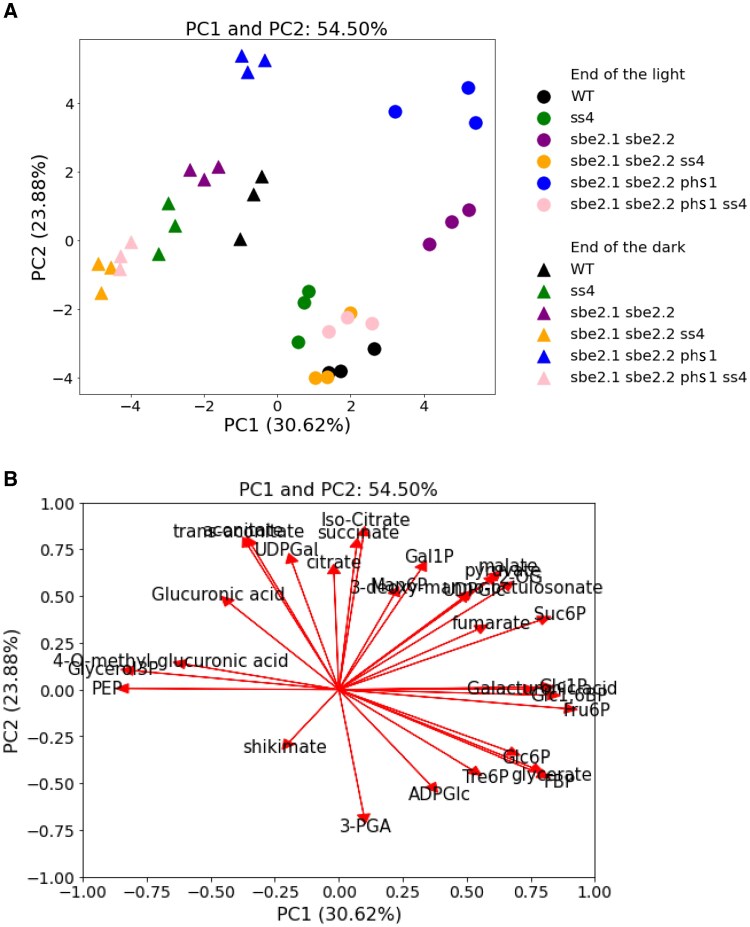
PCA of the metabolite profiles in WT and mutant lines. The analysis was performed using phosphorylated sugars and organic acids. **A)** PCA score plot showing the distribution of genotypes along PC1 (30.62% variance explained) and PC2 (23.88% variance explained), highlighting their separation based on metabolic profiles. Circles and triangles indicate the genotypes under the end-of-light and end-of-dark conditions, respectively. **B)** PCA loading plot illustrating the contribution of individual metabolites to PC1 and PC2 as vectors.

To further investigate the metabolic processes associated with the additional *ss4* mutation, which mitigates the retarded plant growth and elevated maltodextrin levels of the *sbe2.1 sbe2.2 phs1-1* triple mutant, we performed metabolite profiling using 28-d-old rosette leaves of various mutant lines. Samples were collected at the end of the 16-h light and at the end of 8-h dark period. Principal component analysis (PCA) was conducted using the data on phosphorylated sugars and organic acids produced from tricarboxylic acid (TCA) cycle (Fig. 9; Supplementary Data Set 1). Each genotype formed distinct clusters, with clear separation between the end-of-light (circles) and end-of-dark (triangles) conditions along PC1 (Fig. 9A). Notably, at the end of the light period, the *sbe2.1 sbe2.2* double mutant and *sbe2.1 sbe2.2 phs1-1* triple mutant clusters were markedly distant from the WT cluster, with the *sbe2.1 sbe2.2 phs1-1* cluster showing an even greater separation. In contrast, the *sbe2.1 sbe2.2 ss4* and *sbe2.1 sbe2.2 phs1-1 ss4* clusters were positioned much closer to WT (Fig. 9A). The separation was driven primarily along PC2, with a lesser contribution from PC1 (Fig. 9A). Within this cluster, 3 mutants carrying the *ss4* mutation, *sbe2.1 sbe2.2 ss4*, *sbe2.1 sbe2.2 phs1-1 ss4*, and *ss4*, exhibited extremely high ADPGlc levels (approximately 200 nmol/g FW; Supplementary Data Set 1), compared to WT, which contained only 1.2 ± 0.3 nmol/g FW. This suggests that, in addition to elevated ADPGlc, other metabolites are contributing to the distinctive clustering observed in the PCA.

The contribution of individual metabolites to the separation is illustrated in Fig. 9B. Sucrose-6-phosphate (Suc6P), fructose-6-phosphate (Fruc6P), and glucose 1,6-bisphosphate (Glc1,6BP), which are involved in starch and sucrose synthesis and partitioning, emerged as strong positive drivers along PC1. In contrast, phosphoenolpyruvate (PEP) and glycerol-3-phosphate, intermediates of the glycolytic pathway, contributed negatively along PC1. This indicates that these metabolites play a major role in distinguishing the genotypes between the end-of-light and end-of-dark conditions. Along PC2, metabolites associated with the TCA cycle, such as citrate, iso-citrate, aconitate, succinate and malate, were significant positive contributors, while 3-phosphoglycerate (3-PGA), a product of photosynthesis and glycolysis, served as a major negative driver. These analyses suggest that the separation of *sbe2.1 sbe2.2* and *sbe2.1 sbe2.2 phs1-1* from WT is primarily driven by differences in the levels of TCA intermediates. The additional *ss4* mutation partially restored a WT-like metabolic state, as reflected in the closer clustering of *sbe2.1 sbe2.2 ss4* and *sbe2.1 sbe2.2 phs1-1 ss4* with the WT cluster.

## Discussion

The Arabidopsis *sbe2.1 sbe2.2* double mutant exhibits elevated activities of both plastidial and cytosolic PHS isoforms when assayed in the synthetic direction (Fig. 1A). Previous research reported a 4-fold increase in PHS1 and PHS2 activities in the degradative direction in the *sbe2.1 sbe2.2* double mutant (Dumez et al. 2006), suggesting that, as a reversible enzyme, PHS activities in both directions are integrated in this genetic background. Given that both SBEs and PHS1 are localized in chloroplasts, the present study explored the physiological role of PHS1 by introgressing *phs1* loss-of-function mutation into an *sbe2.1 sbe2.2* double mutant of Arabidopsis. The resulting triple mutants, *sbe2.1 sbe2.2 phs1-1* and *sbe2.1 sbe2.2 phs1-2*, were noticeably smaller than the double mutant *sbe2.1 sbe2.2* but remained viable. These unique plant materials provide an opportunity to investigate the function of PHS1 in the *sbe2.1 sbe2.2* background, where normal starch granule formation is blocked but the genes encoding granule initiation are intact.

**Figure 10.**
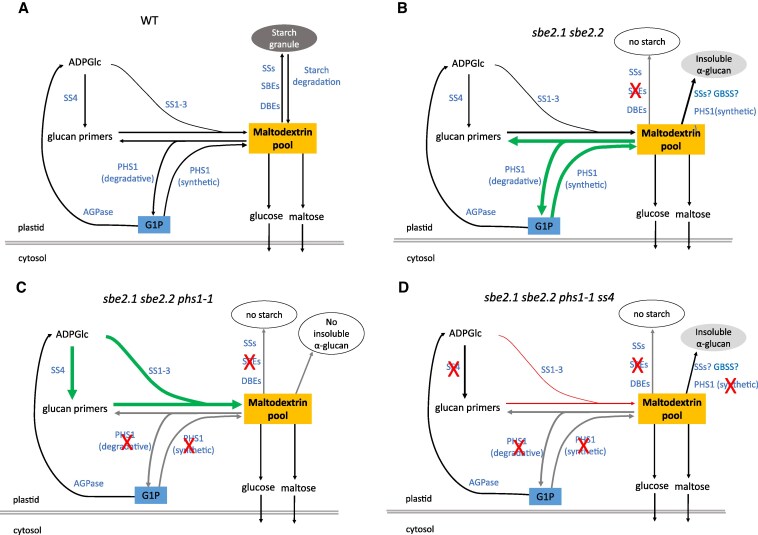
Proposed model for regulation of maltodextrin by PHS1 and SS4 in Arabidopsis leaves. **A)** In WT, the key steps in starch granule initiation and maltodextrin pool regulation are illustrated. Starch granule initiation begins with the formation of the precursor ADPGlc from Glc1P catalyzed by AGPase. SS4 creates α-glucan primers for subsequent α-glucan chain elongation through SS1 to 3, which is then channeled into maltodextrin pool. PHS1's synthetic activity adds Glc1P to short-chain α-glucans, extending them to form longer chain α-glucans within the maltodextrin pool, while its degradative activity breaks down α-glucans phosphorolytically in the maltodextrin pool to release Glc1P. Maltodextrins can either be extended and organized into larger, branched α-glucan structures for starch granule formation through the actions of SSs, SBEs and DBEs, or broken down into maltose and Glc, which is then exported to the cytosol for further metabolism. Starch granule can be degraded and release soluble α-glucans into the maltodextrin pool. **B)** In the *sbe2.1 sbe2.2* double mutant, the SBE mutation impedes starch granule formation, but insoluble linear α-glucans are synthesized, most likely through the combined action of one or more SSs, GBSS, and PHS1 operating in the synthetic direction. High amounts of maltodextrins accumulate, triggering significant increases in PHS1 activity in both its synthetic and degradative pathways (indicated by green arrows). **C)** In the *sbe2.1 sbe2.2 phs1-1* triple mutant, the additional PHS1 mutation caused a compensatory response of elevated SS activities (indicated by green arrows), resulting in even higher amounts of maltodextrins than the *sbe2.1 sbe2.2* double mutant. Insoluble linear α-glucans are undetectable in chloroplasts (indicated by a gray arrow). **D)** In the *sbe2.1 sbe2.2 phs1-1 ss4* mutant, the additional SS4 mutation impairs the starch initiation process, thereby markedly reducing the maltodextrin content possibly by inhibiting other SS isoforms, particularly SS3 (indicated by red arrow). Insoluble linear α-glucans reappear.

The *sbe2.1 sbe2.2* double mutant fails to form typical starch granules in its leaves (Dumez et al. 2006). However, the presence of insoluble linear α-glucans, with an appearance of disorganized starch granule-like structures in ultrastructural analysis, was confirmed through their degradation by α-amylase in situ. These structures were not typical starch granules and, to the best of our knowledge, have not been previously reported. Starch granule formation requires a concerted action of enzymes to promote its crystallization, but the entire process takes a few hours to complete under light conditions (Bürgy et al. 2021). It was observed that clusters of starch granule initials appeared after 15 to 30 min light exposure and are then grouped to form typical starch granules in the Arabidopsis leaf chloroplasts (Bürgy et al. 2021). Thus, we speculate that insoluble linear α-glucans likely accumulated in the stromal pocket region, where starch granules would normally initiate, due to the disruption of further branching in the absence of SBEs. By contrast, no insoluble linear α-glucans were detected in the *sbe2.1 sbe2.2 phs1-1* triple mutant (Fig. 2). Instead, plant growth of the above triple mutant was further retarded, and chloroplast integrity was disrupted. Although soluble α-glucans including maltose and maltodextrin accumulated in the double mutant, they were additionally elevated in the *sbe2.1 sbe2.2 phs1-1* triple mutant. These observations suggest that PHS1 is indispensable for the synthesis of insoluble linear α-glucan in the chloroplasts of the *sbe2.1 sbe2.2* mutant. This process is most likely driven by a direct α-glucan extension reaction, catalyzed by the synthetic activity of PHS1 (Fig. 10). This is consistent with previous reports showing that PHS1 uses Glc1P to produce long-chain maltodextrins in both potato tubers and rice endosperm (Hwang et al. 2016; Flores-Castellanos and Fettke 2023). It is also plausible that the formation of long-chain insoluble α-glucans may result from the cooperative activity of SSs and GBSS, which extend short-chain soluble α-glucans into longer chains (Fig. 10). Given the additionally elevated contents of maltose and maltodextrin in the *sbe2.1 sbe2.2 phs1-1* triple mutant, an alternative scenario in the double mutant could be that PHS1 converts longer-chain maltodextrins into Glc1P and shorter α-glucans in a degradative direction. The shorter α-glucans could then trigger α-glucan chain elongation by SSs, ultimately leading to the accumulation of insoluble α-glucans which are prone to aggregate and form water-insoluble α-glucans (Wu and Sarko 1978; Gidley and Bulpin 1989).

Increased PHS1 activity was also observed in the starch degradation-defective *dpe2* mutant (Malinova et al. 2014). A similar feature to the present study is that the loss of PHS1 in the *dpe2* mutant background led to elevated soluble maltodextrin levels, smaller plant size, and chloroplast degradation (Malinova et al. 2014). The difference between the 2 studies lies in their distinct genetic backgrounds. The *sbe2.1 sbe2.2* double mutant lacks the ability to produce typical starch granules in chloroplasts, but its starch initiation mechanism and cytosolic maltose metabolism pathway remains functional. In contrast, in the *dpe2* mutant, starch synthesis is functional but starch degradation is perturbed because the mutant cannot efficiently metabolize maltose, the main product of starch degradation, when it is exported into the cytosol. Although the single *phs1* mutant does not affect starch turnover or structure (Zeeman et al. 2004), the role of PHS1 in maltodextrin turnover becomes more prominent when the mutation occurs in specific genetic backgrounds that lack normal starch synthesis (e.g. the *sbe2.1 sbe2.2* double mutant) or degradation (e.g. the *dpe2* mutation). It is reasonable to propose that PHS1 acts as a buffer, coordinating its synthetic and degradative activities to regulate chloroplast maltodextrin levels during diurnal starch turnover.

Further investigation revealed that the *sbe2.1 sbe2.2 phs1-1* triple mutant possessed more than 2-fold higher total SS activity compared to the double mutant. This upregulation of SS activity was likely contributed by all SS gene family members, including GBSS, SS1, SS2, SS3, and SS4, as indicated by their protein levels and enzymatic activities (Fig. 4). Consistent with higher measurable SS activities, the *sbe2.1 sbe2.2 phs1-1* triple mutant exhibited consistently lower ADPGlc concentrations than the double mutant throughout a 24-h diurnal period, suggesting increased ADPGlc utilization by SSs in the *sbe2.1 sbe2.2 phs1-1* triple mutant (Fig. 4). Given that SS4 plays a crucial role in starch granule initiation (Roldan et al. 2007; Szydlowski et al. 2009; Crumpton-Taylor et al. 2013; Ragel et al. 2013; Malinova et al. 2018; Merida and Fettke 2021), and an interaction between SS4 and PHS1 has been proposed in regulating starch granule number and morphology (Malinova et al. 2017), it might be expected that SS4 is indispensable in the *sbe2.1 sbe2.2 phs1-1* triple mutant. Surprisingly, our results contradict this hypothesis (Fig. 5 and Table 2). First, the combined mutation of *ss4* with *sbe2.1 sbe2.2 phs1-1* improved plant growth and significantly increased FW, compared to the *sbe2.1 sbe2.2 phs1-1* triple mutant under a 16-h photoperiod. Second, introgression of the *ss4* mutation into the triple mutant restored the formation of insoluble linear α-glucans in the chloroplasts of the *sbe2.1 sbe2.2 phs1-1 ss4* mutant. Third, maltose and maltodextrin contents were strongly decreased in *sbe2.1 sbe2.2 phs1-1 ss4* compared to the *sbe2.1 sbe2.2 phs1-1* triple mutant (Table 2). Finally, chloroplast integrity in *sbe2.1 sbe2.2 phs1-1 ss4* was largely recovered by the additional loss of SS4 (Fig. 6). These results collectively demonstrated that the defective phenotypes observed in the *sbe2.1 sbe2.2 phs1-1* triple mutant were mitigated by the loss of SS4.

SS4 is believed to produce short linear glucan chains, typically ranging from DP3 to 7, necessary for the initial formation of starch granules (Szydlowski et al. 2009). Based on this, we hypothesize that the *ss4* mutation in the *sbe2.1 sbe2.2 phs1-1* triple mutant background prevents the formation of short-chain α-glucan primers, which are normally used for elongation by SS1 to 3, leading to a significant decrease in total soluble α-glucan content. This notion is further supported by the fact that in the *sbe2.1 sbe2.2 phs1-1 ss4* mutant, the protein levels of SS1, SS2, and SS3 were all decreased compared to *sbe2.1 sbe2.2 phs1-1* (Fig. 8A). Moreover, the decrease in DPE2 enzyme activity (Fig. 8D) linked to the *ss4* mutation implies that the latter mutation lowered the availability of soluble α-glucans, particularly maltose, the usual substrate for DPE2. The precise localization of maltodextrins in the *sbe2.1 sbe2.2 phs1-1* triple mutant remains unclear. Given that over 80% of maltodextrin is typically in the form of maltose, which can be exported to the cytosol for further metabolism (Niittyla et al. 2004; Weise et al. 2004), it is nonetheless possible that when maltose concentrations become excessively high, a portion is redirected back into the chloroplasts (Stettler et al. 2009). Since high maltodextrin concentrations are thought to create osmotic pressure on chloroplast envelope and thylakoid membranes (Stettler et al. 2009; Malinova et al. 2014), it is plausible that the decreased levels of maltodextrins helped maintain chloroplast membrane integrity, facilitating the formation of stromal pockets that support the subsequent production of insoluble linear α-glucans. ADPGlc, the key precursor in starch synthesis, plays a crucial role in influencing overall plant growth and chloroplast function (Zeeman et al. 2010). PCA analysis indicates that other than ADPGlc, there are also other metabolites contributing to the observed clustering (Fig. 9). The low maltodextrin content in *sbe2.1 sbe2.2 phs1-1 ss4*, *sbe2.1 sbe2.2 ss4*, *ss4* single mutant, and WT, corresponded closely with the PCA cluster, particularly at the end of the 16-h light period (Fig. 9A). These results suggest that decreased maltodextrin levels, in combination with elevated ADPGlc, may be major factors driving the changes in TCA cycle metabolites observed in these lines.

In the *sbe2.1 sbe2.2* double mutant background where the chloroplast envelope and thylakoid membranes remained organized, the additional loss of SS4 further increased the amount of insoluble linear α-glucans (0.62 vs. 1.24 mg/g FW) (Table 2). Insoluble linear α-glucans were clearly evident in the *sbe2.1 sbe2.2 phs1-1 ss4* mutant whereas no such glucans were observed in the *sbe2.1 sbe2.2 phs1-1* mutant. This likely results from a reduction in α-amylase activity (Supplementary Fig. S8), possibly due to a decrease in AMY3 (Seung et al. 2016), thereby limiting glucan primer degradation and promoting the synthesis of insoluble α-glucans in the quadruple mutant which also carries the *phs1* mutation. The data show that the *ss4* mutation strongly decreased the levels of maltose and maltodextrin to similar amounts in both the double and triple mutant (*sbe2.1 sbe2.2 phs1-1*) backgrounds by the end of the light period, despite the triple mutant (*sbe2.1 sbe2.2 phs1-1*) initially containing 30% more maltose and 150% more maltodextrin than the double mutant. However, loss of SS4 in the *sbe2.1 sbe2.2 phs1-1* triple mutant restored chloroplast membrane integrity but only increased the amount of insoluble α-glucan to 0.58 ± 0.04 mg/g FW, which is half of that observed in the *sbe2.1 sbe2.2 ss4* mutant (1.24 ± 0.06 mg/g FW). This suggests that PHS1 could account for at least half of the insoluble linear α-glucans synthesized in the *sbe2.1 sbe2.2 ss4* mutant (Fig. 10B).

To rationalize the impacts of PHS1 and SS4 on maltodextrin regulation, we propose a model (Fig. 10) that attempts to explain the metabolic changes in mutants carrying combinations of *phs1* and *ss4* mutations within the *sbe* double mutant background. In WT (Fig. 10A), SS4 contributes to the synthesis of short, soluble α-glucan primers during the early stages of starch biosynthesis for the subsequent elongation by other SSs (SS1, SS2, and SS3), to generate maltodextrins. The synthetic direction of PHS1, which utilizes Glc1P as a substrate, contributes to the maltodextrin pool, while its degradative pathway breaks down maltodextrin to release Glc1P. Maltodextrins can either be extended and organized into larger, branched α-glucan structures for starch granule formation, or broken down into maltose (by β-amylase) and Glc (by DPE1), which is then transported to the cytosol for further metabolism. However, PHS1's role in WT appears to be minimal. In the *sbe2.1 sbe2.2* double mutant (Fig. 10B), the failure to form starch granules leads to the accumulation of maltodextrin and maltose, which in turn trigger significant increases in PHS1 activity in both its synthetic and degradative pathways. Consequently, insoluble linear α-glucans were synthesized, most likely through the combined action of one or more SSs, GBSS, and synthetic direction of PHS1. In the *sbe2.1 sbe2.2 phs1-1* triple mutant (carrying the *phs1* mutation; Fig. 10C), all SSs (SS1 to 4) are upregulated compared to the double mutant, possibly as a compensatory response to sustain the maltodextrin pool caused by deficiency in PHS1, thus resulting in even higher maltodextrin and maltose levels than the double mutant. No insoluble linear α-glucans in the *sbe2.1 sbe2.2 phs1-1* mutant were generated likely due to disrupted chloroplast membrane integrity. In the *sbe2.1 sbe2.2 phs1-1 ss4* mutant, the additional loss of SS4 severely disrupts the starch granule initiation process, reducing the availability of α-glucan primers and subsequently decreasing maltodextrin and maltose levels, including by suppressing SS protein levels and activity, particularly that of SS3 (Fig. 8, A and B), resulting in partially restored plant growth and re-generation of insoluble α-glucans. The partially reversed phenotype associated with the additional *ss4* mutation was also reflected in metabolite profiling, revealing that TCA cycle metabolites are the primary drivers conferring a WT-like metabolic state in *sbe2.1 sbe2.2 ss4* and *sbe2.1 sbe2.2 phs1-1 ss4* mutants (Fig. 9). The distinctly elevated levels of TCA cycle intermediates in the *sbe2.1 sbe2.2 phs1-1* mutant suggest a greater flux through the TCA cycle, possibly to meet increased energy production demands, but also as an alternative sink for carbon in the absence of starch granule initiation. The loss of SS4 in the *sbe2.1 sbe2.2* double mutant suppresses PHS1 activity compared to the double mutant (Fig. 8E), consistent with decreased soluble α-glucan availability in the *sbe2.1 sbe2.2 ss4* mutant. We propose that PHS1 plays a vital role in metabolizing maltodextrins in the chloroplast and interacts dynamically with SS4 in various genetic contexts to ensure efficient maltodextrin metabolism. This perspective is particularly relevant in the specific genetic background where starch granule formation is disrupted or under stress conditions, further underscoring PHS1's buffering role in managing the maltodextrin pool.

## Materials and methods

### Plant material and growth conditions

All Arabidopsis (*A. thaliana*) plants used in this study were of the accession Columbia. The T-DNA insertion mutants *sbe2.1* (SALK_048089), *sbe2.2* (SALK_107255), *phs1-1* (SALK_055562), *phs1-2* (GK-257A06, ABRC stock no. CS731991), and *ss4* (CS2103513) were obtained from the ABRC at Ohio State University (Alonso et al. 2003; http://abrc.osu.edu). Homozygous mutants were verified using 2 gene-specific primers (90974 and 90975 for SALK_048089, 90972 and 90973 for SALK_107255, 98125 and 98126 for SALK_055562, 98122/98123 for GK-257A06 [CS731991], 105859/105860 for CS2103513) and border primers (LBa1 and 90975 for SALK_048089, LBa1 and 90973 for SALK_107255, LBa1 and 98126 for SALK_055562, 98122/8474 for GK-257A06 [CS731991], 8474/105860 for CS2103513). The double mutant *sbe2.1 sbe2.2* was generated in our previous study (Liu et al. 2016). The *sbe2.1 sbe2.2 phs1-1* and *sbe2.1 sbe2.2 phs1-2* triple mutants were generated using *sbe2.1 sbe2.2* as the pollen recipient and *phs1-1* or *phs1-2* as the pollen donor, respectively. The *sbe2.1 sbe2.2 ss4* and *sbe2.1 sbe2.2 phs1-1 ss4* mutants were generated using *sbe2.1 sbe2.2* and *sbe2.1 sbe2.2 phs1-1* as the pollen recipient, respectively, and *ss4* as the pollen donor. After selfing of the resulting F1 plants, homozygous mutants in the F2 progeny were identified by PCR genotyping. For comparison, these mutants were always grown along with the WT. In this study, all plants were grown at 18 to 22 °C with a 16-h light/8-h dark diurnal cycle, at 150 *µ*mol m^−2^ s^−1^ irradiance (supplied by white fluorescent tubes), and <60% humidity.

### Protein extraction and measurement

Plant materials were frozen in liquid nitrogen. Total soluble protein was extracted by mixing ground powders of rosette leaves and extraction buffer (50 mM Tris–HCl [pH 8.0], 1 mm Na_2_-EDTA, 150 mM NaCl, 1% [w/v] ProteaseArrest protase inhibitor cocktail, Sigma) followed by centrifugation at 16,000 × *g* for 5 min at 4 °C. Protein content was determined using the Bio-Rad Quick Start Bradford reagent (www.biorad.com).

### In-gel zymogram assays

For each zymogram enzyme assay, samples of 100 *µ*g protein were electrophoresed at 90 V for 3 h at 4 °C. Activities were visualized after staining gels with Lugol's solution. PHS activity was performed using 7% (w/v) nondenaturing polyacrylamide gels containing 0.3% (w/v) maltodextrin, followed by incubation in a buffer (100 mm sodium citrate, pH 5.6; 20 mm Glc1P; 0.1% [w/v] glycogen from oyster; 1 mm Na_2_-EDTA; 1 mm DTT) at 28 °C overnight (Wang et al. 2022). SS activity was visualized in 5% (w/v) continuous nondenaturing polyacrylamide gels containing 0.3% (w/v) glycogen, followed by incubation in a buffer (50 mm glycylglycine; 100 mm sodium citrate; 20 mm DTT; 5 mm MgCl_2_; 0.5 mg/mL BSA; 2 mm ADPGlc; pH 9) at 28 °C for 48 to 72 h. DPE1 and DPE2 activities were visualized using 7% (w/v) nondenaturing polyacrylamide gels containing 1% (w/v) and 0.1% (w/v) glycogen, respectively. The incubation buffer contained 100 mm Tris-HCl (pH7.0), 1 mm MgCl_2_, 1 mm Na_2_-EDTA, 1 mm DTT, 10 mm maltotriose for DPE1 (Critchley et al. 2001), and 20 mm maltose for DPE2 (Li et al. 2022). Gels were incubated in reagents at 28 °C overnight (16 h). Alpha-amylase activity was detected using 7% (w/v) nondenaturing polyacrylamide gels containing 0.3% (w/v) maltodextrin.

### Quantitative enzyme assays

Soluble protein extracts were desalted using NAP-5 Sephadex Columns G-25 (DNA Grade, GE Healthcare). SS and ADPGlc pyrophosphorylase (AGPase) assays were conducted according to Kulichikhin et al. (2016). Briefly, SS activity was measured as the α-glucan-primer dependent release of ADP from ADPGlc, with ADP production being monitored spectrophotometrically at 340 nm by coupling to reduction of NADP^+^ via pyruvate kinase, hexokinase, and glucose-6-phosphate dehydrogenase. AGPase activity was determined in the reverse direction as the pyrophosphate-dependent production of Glc1P from ADPGlc. Glc1P production was monitored as above, by coupling to NADP^+^ reduction via phosphoglucomutase and glucose-6-phosphate dehydrogenase.

β-Amylase and D-enzyme activities were performed according to Zeeman et al. (1998). To determine β-amylase activity, leaf extract containing 100 *µ*g protein was mixed with 50 mm sodium acetate (pH 5.6), 5 mm Na_2_-EDTA, 5 mm DTT, and 5 mg/mL potato starch for 45 min at 25 °C. The resultant maltose was hydrolyzed by maltase to release Glc for quantification (Smith and Zeeman 2006). To measure D-enzyme activity, the reaction mixture containing 50 mm sodium acetate (pH 6.5), 50 mm maltotriose, and 100 *µ*g protein was incubated at 30 °C for 30 min, and the resultant Glc was determined (Wang et al. 2020b; Smith and Zeeman 2006).

### Immunoblotting

Leaf extracts containing 100 *µ*g protein were mixed with SDS loading buffer, boiled for 5 min, and loaded onto 12% (w/v) acrylamide gels. Electrophoresis was carried out at 120 V for 100 min at room temperature. For immunoblotting, the separated proteins were transferred onto nitrocellulose membranes by electroblotting, and then, the membrane was blocked with 1 × TBS containing 1.5% (w/v) BSA on a table shaker for 15 min. Purified peptide-specific antibodies raised against maize PHS1, GBSS, or SS4 and against Arabidopsis SS1, SS2, or SS3 were used at a 1:5,000 dilution in 1 × TBS containing 1.5% (w/v) BSA. Goat anti-rabbit IgG conjugated with alkaline phosphatase (Sigma) served as the secondary antibody (Wang et al. 2022). An aliquot of 5 to 10 mL of alkaline phosphatase substrate solution (BCIP/NBT) was applied to the membranes for development, resulting in the formation of an insoluble colored product that allowed the visualization of the target protein bands.

### In situ leaf α-glucan digestion

Four-week-old rosette leaves were collected from the plants at the end of the 16-h light period. Ethanol (80%, v/v) was immediately added to remove chlorophylls at 95 °C for 5 to 10 min until the leaves were completely decolored. Each piece of leaf was placed on the microscope slide, to which 30 units of α-amylase (Megazyme) in 200 mm sodium acetate buffer (pH 5.2) was added and then placed in a sealed plastic box at 37 °C overnight.

### RT-PCR

Total RNAs were extracted from leaf tissues of 4-wk-old mutants and WT plants using RNeasy mini plant kit (Qiagen) and cDNAs synthesized with a QuantiTect Reverse Transcription Kit (Qiagen). To detect the transcripts, PCR was performed using the following primers: SS1-F1/SS1-R1 for *SS1*, SS2-F1/SS2-R1 for *SS2*, SS3-F1/SS3-R1 for *SS3*, SS4-F1/SS4-R1 for *SS4*, SS5-F1/SS5-R1 for *SS5*, and GBSS-F1/GBSS-R1 for *GBSS*, with 27 amplification cycles (98 °C, 10 s; 56 °C, 15 s; 72 °C, 20 s; Supplementary Table S1). Arabidopsis 18S rRNA served as the internal control, amplified using 20 PCR cycles.

### TEM and light microscopy

Four-week-old rosette leaves were embedded in Spurr's resin according to Wang et al. (2020a). Mesophyll cell chloroplasts were viewed on an FEI Tecnai G2 F20 TEM, with a Gatan 4 K CCD camera, and Gatan Digital Micrograph (Gatan Inc., Pleasanton, CA) software used to record images. Semi-thin sections (100 nm) were stained with toluidine blue (0.5%, w/v) and photographed using an advanced light microscope (Nikon Eclipse Ti2, Leica DM 5000B).

### Metabolite extraction and determination

Starch and insoluble α-glucans were quantified according to Wang et al. (2020b). Soluble sugars (Glc, fructose, sucrose, maltose, and maltodextrin) were extracted using 80% (v/v) ethanol and enzymatically processed to release Glc for quantification (Stitt et al. 1989). The Glc generated from each reaction was measured and used to calculate the content of the corresponding sugars according to Smith and Zeeman (2006).

Phosphorylated intermediates and organic acids were extracted as described in Lunn et al. (2006). Briefly, 15 to 20 mg of frozen leaf tissue was extracted with 350 *µ*L of an ice-cold chloroform/methanol mixture (3:7, v/v). The sample was kept at −20 °C for 2 h, followed by the addition of 350 *µ*L of ice-cold water and thorough mixing. After centrifugation at 13,000 × *g* for 10 min, the upper aqueous phase was transferred to a new Eppendorf tube and dried using a centrifugal vacuum. Samples were analyzed by LC-MS/MS as described by Lunn et al. (2006), with modifications (Figueroa et al. 2016).

### Maltodextrin profile

Total soluble sugars used for quantification were mixed with 1 mL of 10 mm sodium acetate (pH 3.5) and filtered through a 0.45 *µ*m syringe filter. A 20 *µ*L aliquot was analyzed using a Dionex ICS 3000 High-Performance Anion-Exchange Chromatography system with a PA-100 column (Thermo Fisher Scientific). Peaks were identified using maltose, maltotriose, and maltodextrin standards, and their percentages were calculated from peak areas (Bertoft et al., 2008; Annor et al. 2014). The nmol amounts of DP3-17 were determined by multiplying their percentage by the total maltodextrin content in each sample.

### Separation of sugars using TLC

A small aliquot of total soluble sugars extracted from 400 mg (FW) leaves was separated on silica G60 TLC plates (Merck) using a solvent system of butanol: acetone: water (40:50:10, by volume) for 2.5 h. The bands were developed by spaying with ethanol containing 0.5% (w/v) a-naphthol and 5% (v/v) sulfuric acid, followed by heating for 10 min at 120 °C (Robyt and Mukerjea 1994).

### Statistical analyses

Statistical analyses were performed in Anaconda3 (www.anaconda.com) with Python version 3.7.4 using either PCA function (from Python package of sklearn) or ANOVA function (Sums of Squares Type II) followed by the Tukey's Honest Significant Differences posttest (from Python package of statsmodels). Additional details are provided in the figure legends.

### Accession numbers

Sequence data from this article can be found in the Arabidopsis Genome Initiative database under the following accession numbers: *SBE2.1* (At2g36390); *SBE2.2* (At5g03650); *PHS1* (At3g29320); *SS1* (At5g24300); *SS2* (At3g01180); *SS3* (At1g11720); *SS4* (At4g18240); *SS5* (At3g11340); and *GBSS* (At1g32900).

## Supplementary Material

kiaf216_Supplementary_Data

## Data Availability

The data underlying this article are available in the article and in its online supplementary materials.

## Supplementary data

The following materials are available in the online version of this article.

**Supplementary Figure S1.** Identification of T-DNA insertion mutant lines *phs1-1* and *phs1-2*.

**Supplementary Figure S2.** Growth phenotypes.

**Supplementary Figure S3.** Enzyme activity analyses.

**Supplementary Figure S4.** RT-PCR analyses.

**Supplementary Figure S5.** Separation of sugars using TLC.

**Supplementary Figure S6.** Representative chromatographic traces.

**Supplementary Figure S7.** Zymogram analysis of DPE1 activity in WT and the *dpe1* mutant.

**Supplementary Figure S8.** Zymogram analysis of α-amylase activity in mutants carrying the *ss4* mutation.

**Supplementary Table S1.** Primer sequences used in this study.

**Supplementary Data Set 1.** A complete list of the measured metabolites from the 28-d-old rosette leaves collected at the end of 16-h light and the end of 8-h dark period, respectively.

## Dive Curated Terms

The following phenotypic, genotypic, and functional terms are of significance to the work described in this paper:

DPE2 Gramene: AT2G40840
DPE2 Araport: AT2G40840

SDS Gramene: AT1G14750
SDS Araport: AT1G14750

PHS1 Gramene: At3g29320
PHS1 Araport: At3g29320

SS2 Gramene: AT1G74020
SS2 Araport: AT1G74020

SS3 Gramene: AT1G74000
SS3 Araport: AT1G74000

starch CHEBI: CHEBI:28017

protein CHEBI: CHEBI:36080

AMY3 Gramene: AT1G69830
AMY3 Araport: AT1G69830

DPE1 Gramene: AT5G64860
DPE1 Araport: AT5G64860

PCR Gramene: Polymerase Chain Reaction
PCR Araport: Polymerase Chain Reaction

PTST2 Gramene: AT1G27070
PTST2 Araport: AT1G27070
