# Supplementary material for: Plastidial starch phosphorylase regulates maltodextrin turnover during starch granule initiation in Arabidopsis leaves
Source: Plant Physiol. 2025 Jun 3;198(2):kiaf216. doi: 10.1093/plphys/kiaf216 (PMC12203538; doi:10.1093/plphys/kiaf216)
Supplement: kiaf216_Supplementary_Data [file kiaf216_supplementary_data.zip › Supplementary Data.pdf]

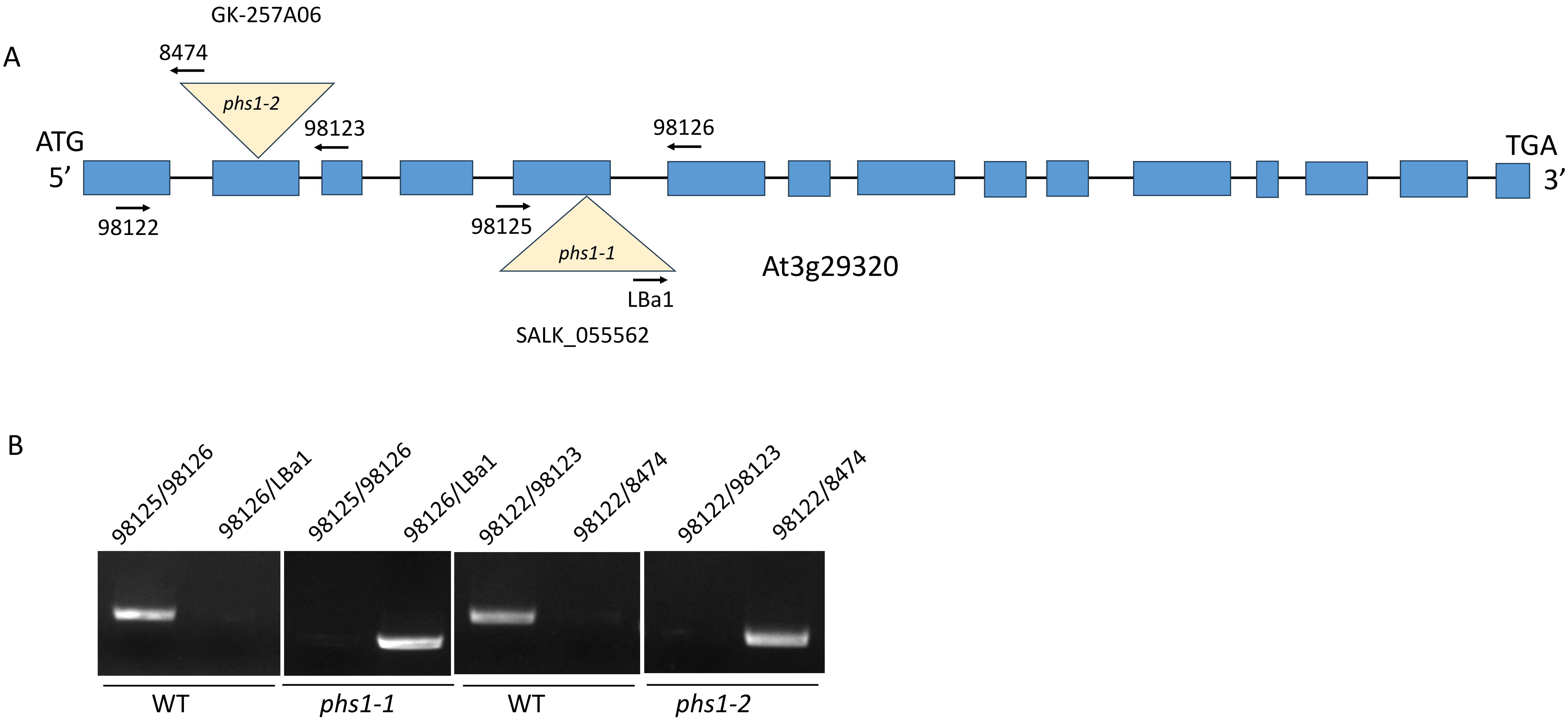

Supplementary Figure S1. Identification of T-DNA insertion mutant lines *phs1-1* and *phs1-2*. A, Schematic representation of the T-DNA insertion sites in *phs1* single mutant alleles. The insertion sites were illustrated by the triangles. Blue boxes represent exons and black lines indicate introns. Arrows denote the primers used for the characterization of homozygous mutant. SALK_055562 corresponds to the *phs1-1* mutant (SALK line), GK-257A06 (ABRC No. CS731991) represents the *phs1-2* mutant (GABI-Kat line). **B**, Identification of T-DNA insertion lines through PCR analysis. Primers LBa1 and 8474 are flanking primers for SALK and GABI-Kat lines, respectively, with specific primer combinations indicated for each genotype.

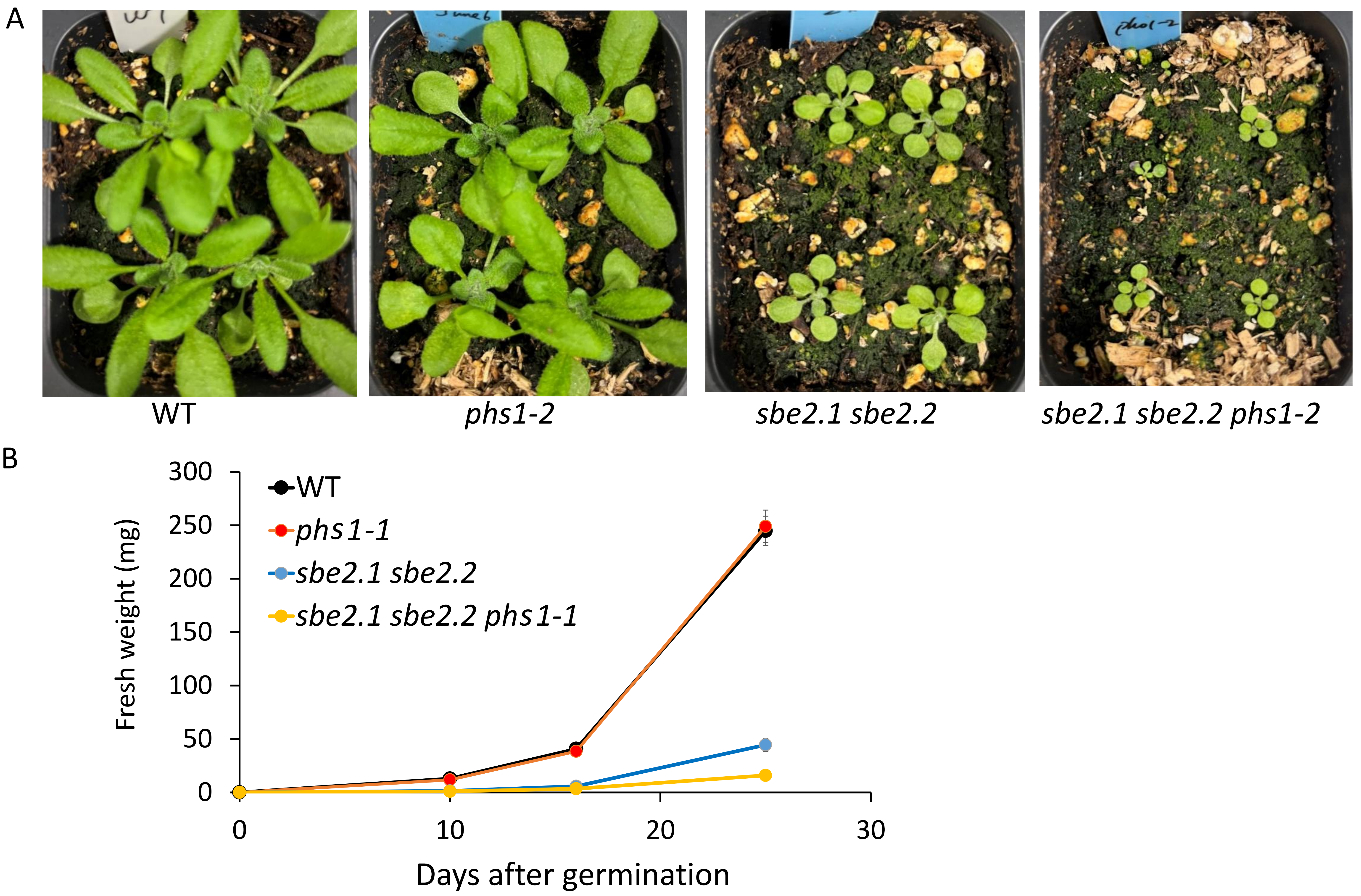

Supplementary Figure S2. Growth phenotypes. **A**, Phenotype of *phs1-2* mutant, *sbe2.1 sbe2.2* double mutant, *sbe2.1 sbe2.2 phs1-2* triple mutant and the WT plant. Images were captured of plants at 18-day post-germination. **B**, FW of the above ground tissues of *phs1-1*, *sbe2.1 sbe2.2*, *sbe2.1 sbe2.2 phs1-1* and WT plants. Values are means ± SE (n = 15-20).

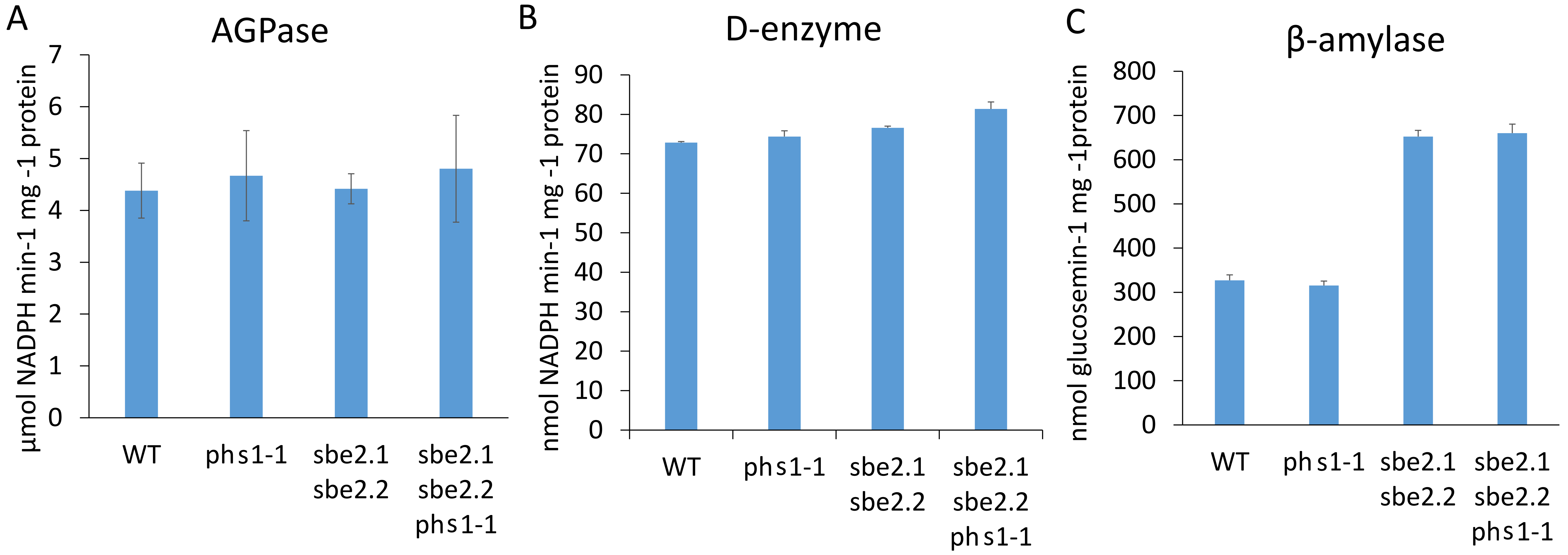

Supplementary Figure S3. Enzyme activity analyses. Four-week-old rosette leaves were collected at the end of the 16-hour light period, and 100 µg protein extract was used for each optimized assay. **A**, AGPase; **B**, D-enzyme; **C**, β-amylase. Values represent means ± SE (n = 3, leaves were pooled from at least 10 plants).

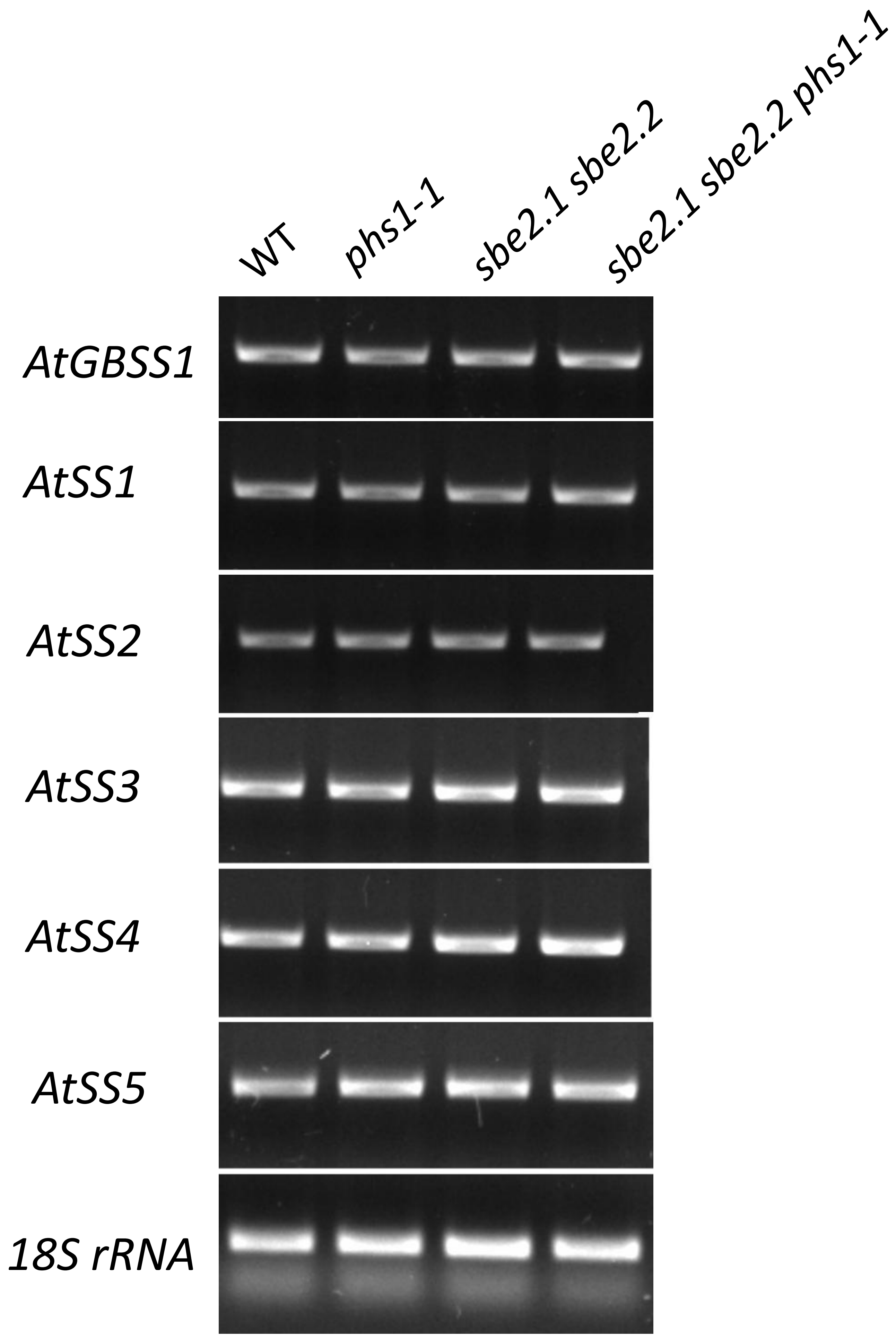

Supplementary Figure S4. RT-PCR analyses. Expression levels of starch synthase genes *AtGBSS1*, *AtSS1*, *AtSS2*, *AtSS3*, *AtSS4* and *AtSS5* were compared. Total RNAs were extracted from 25d-old rosette leaves and *18S rRNA* was used as a reference gene.

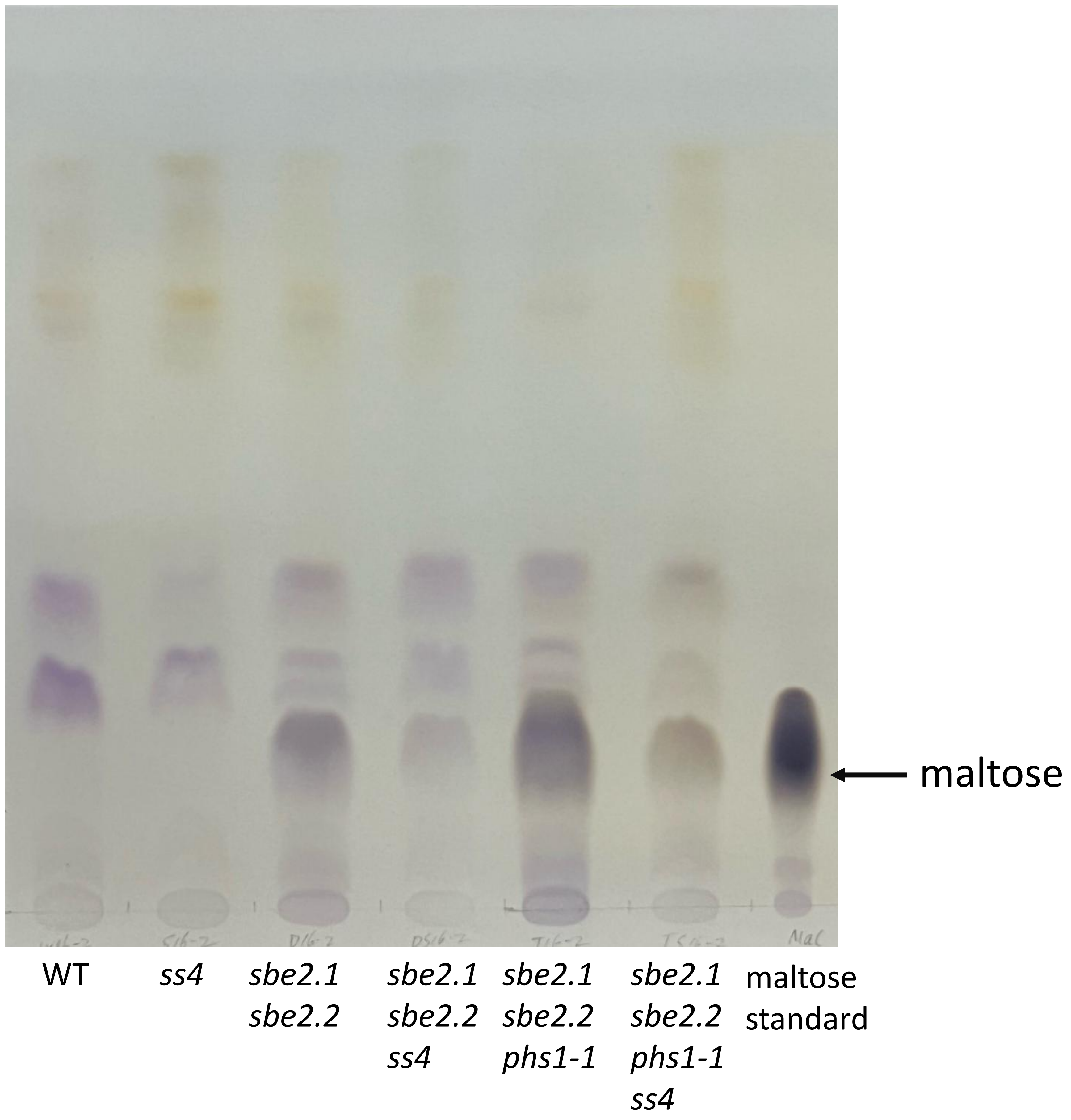

Supplementary Figure S5. Separation of sugars using TLC. A small aliquot of total soluble sugars was separated on silica G60 TLC plates (Merck) using a solvent system of butanol: acetone: water (40:50:10, v/v/v). Maltose standard was used as a reference. The sugar extracts were from the leaf samples collected at the end of 16-h light period.

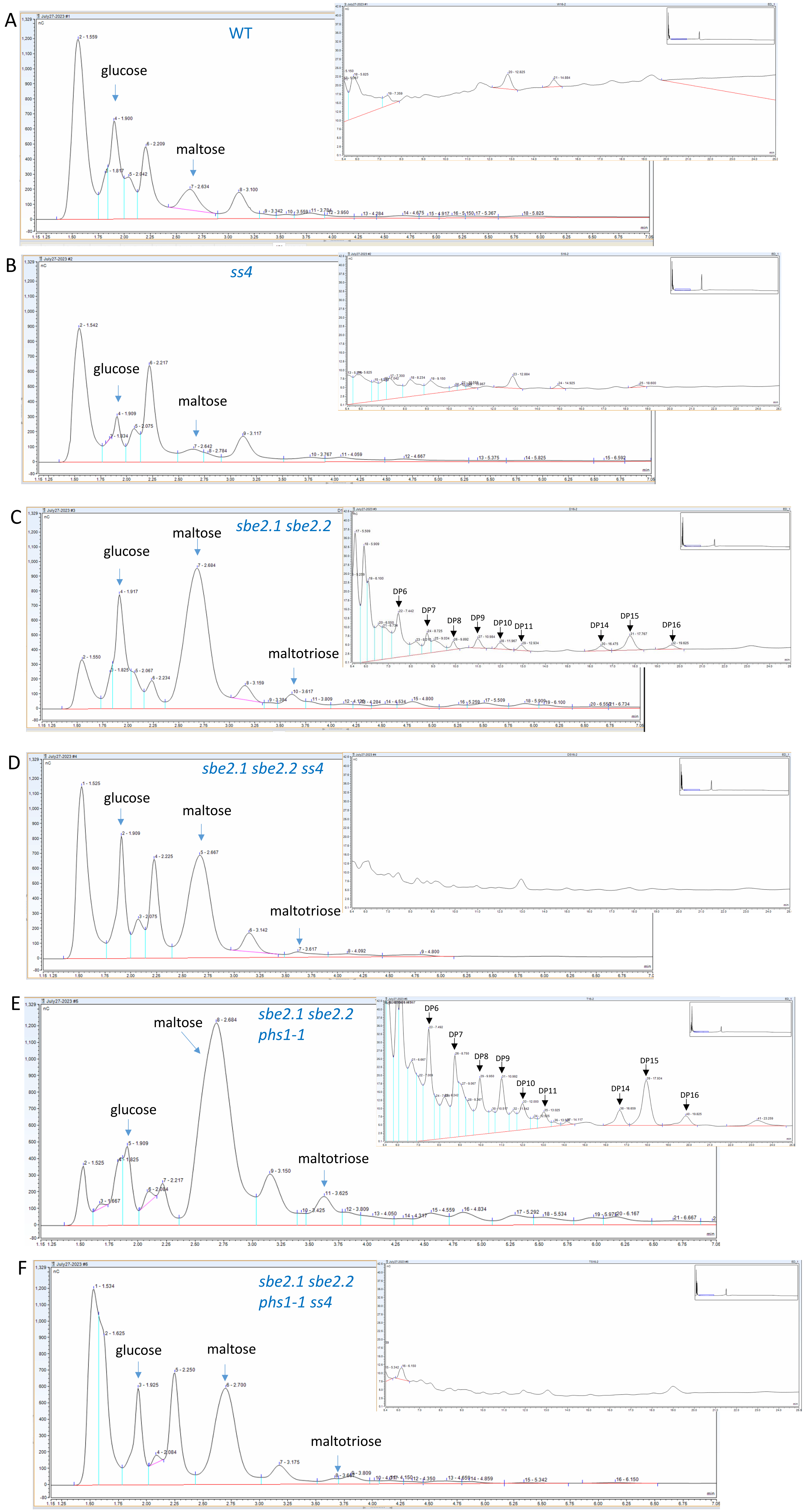

Supplementary Figure S6. Representative chromatographic traces. A, WT; **B**, *ss4* single muant; **C**, *sbe2.1 sbe2.2* double mutant; **D**, *sbe2.1 sbe2.2 ss4*; **E**, *sbe2.1 sbe2.2 phs1-1* triple mutant; **F**, *sbe2.1 sbe2.2 phs1-1 ss4*. Insets in each panel provide an enlarged diagram showing the peaks during the extension time of 5.4-22 min. Glc, maltose and maltotriose are indicated by blue arrows, and long-chain maltodextrins (DP6-16) were denoted by dark arrows in (**C**) and (**E**). The y-axis represents charge in nanoCoulombs (nC), which reflects the amount of electrical charge collected over time and serves as a quantitative indicator of carbohydrate content in the sample. The x-axis indicates the retention time in minutes.

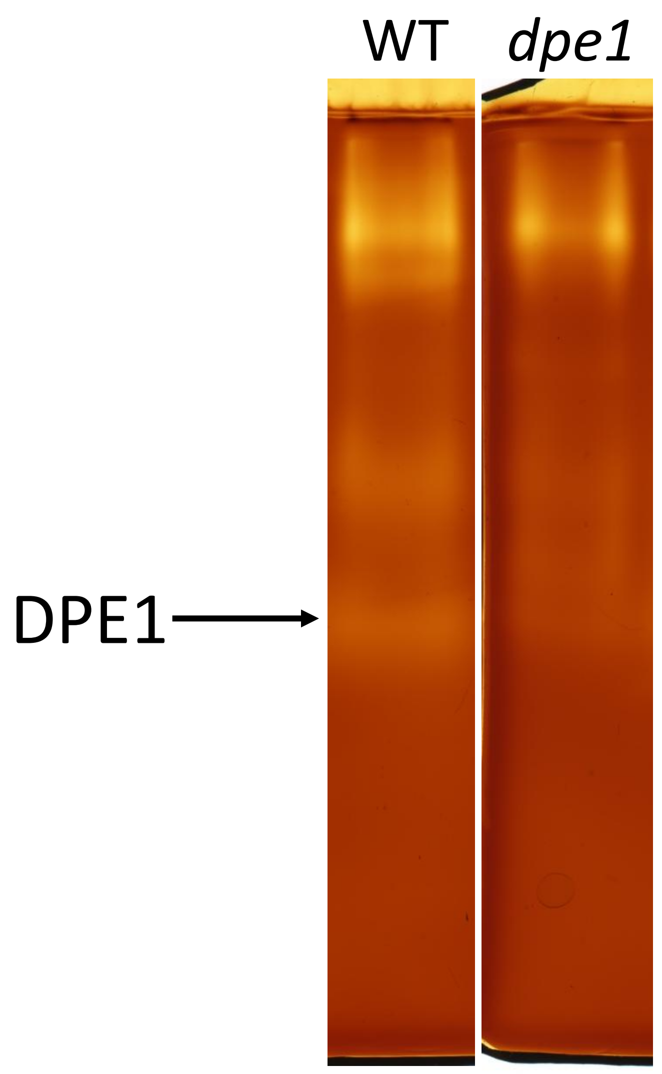

Supplementary Figure S7. Zymogram analysis of DPE1 activity in WT and the *dpe1* mutant. Maltotriose was used as the substrate. Four-week-old rosette leaves were harvested at the end of the 16-h light period.

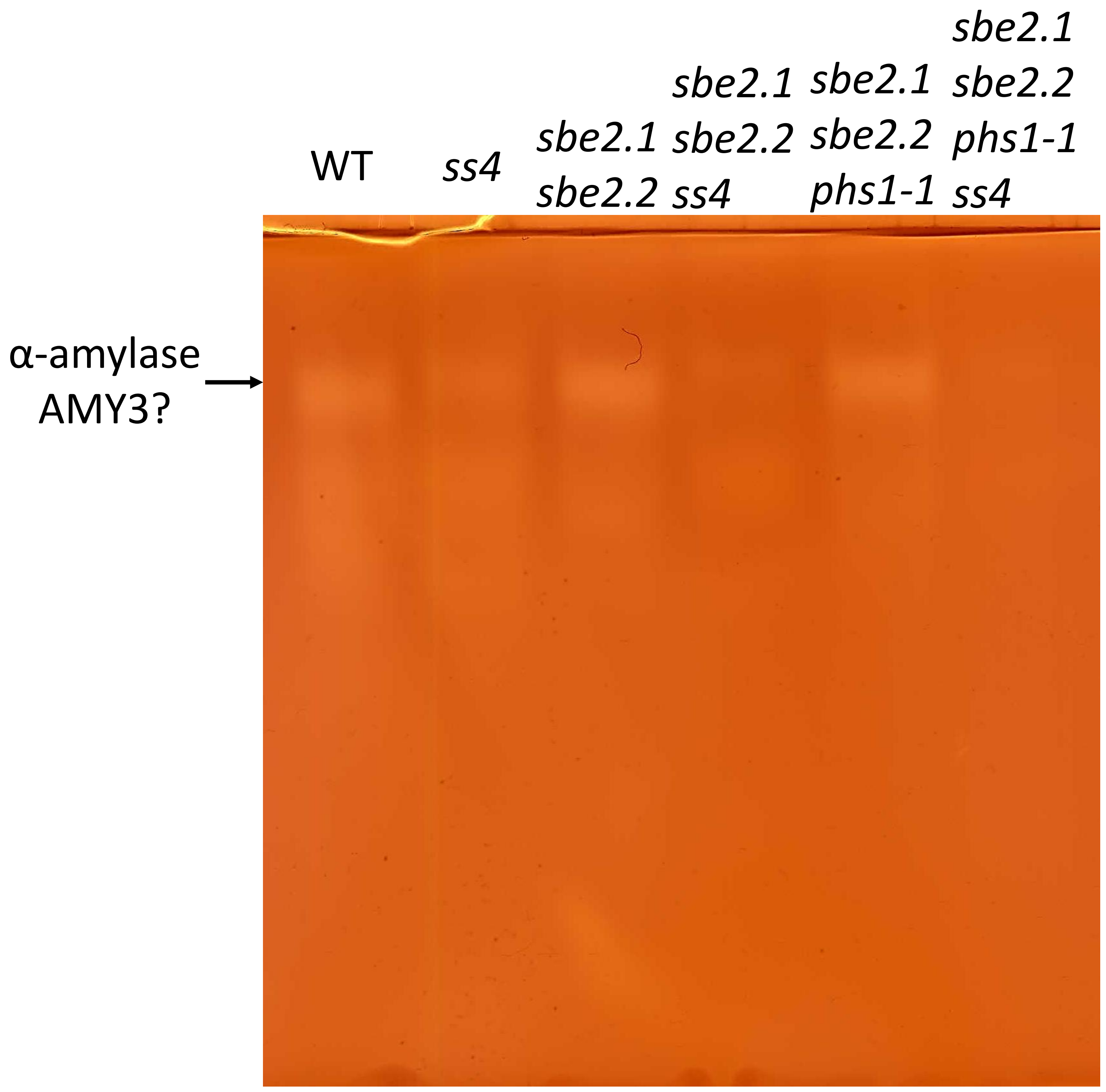

Supplementary Figure S8. Zymogram analysis of α-amylase activity in mutants carrying the *ss4* mutation. Maltodextrin was used as the substrate. Four-week-old rosette leaves were harvested at the end of the 16-h light period. α-amylase activity was determined according to Seung *et al*. (2016).

Supplemental Table S1. Primer sequences used in this study

Primer name	Primer sequence (5'-3')
90974	TGT ATT CTT CCA GCC ACC ATC
90975	ATA TGC GTT CAA ACA TCC TCG
90972	ACA CAC ACC GTG CTA CTC ATG
90973	TTG TTT TGC TAA CTT CTG CCG
98125	ACT GCG CAA AGT GTT AGA GAT GC
98126	ACC TTT TCA GCG TTG AAT AGA G
98122	GTA ACT GAT TCG TAT TCC TTG GGA
98123	CCG GAT AAT TCA AAG TTG CCA TGG
105859	TAC CGC GTC TGC TTC AGA ATT G
105860	TGA CGG AAT GAT GAA CAA ATC G
LBa1	TGG TTC ACG TAG TGG GCC ATC G
8474	ATA ATA ACG CTG CGG ACA TCT ACA TTT T
SS1-F1	TAA GGA TGC AAG AAG CAT TC
SS1-R1	CTA TCT TTC GAC AAG GGA GT
SS2-F1	TGT GAA CGG TAT CGA TAC AC
SS2-R1	CAA ATC CTG TGT CAT TCC TC
SS3-F1	TGA CAT TTG CAG ACA AAG CC
SS3-R1	GAG CTC AAG ATA CTC AAG TG
SS4-F1	ACT TAT GCA CAA GAA GTT CG
SS4-R1	GTA GAG TTC TTC GTA TTG TG
SS5-F1	TGT ATT TCC TCT TGA TGT CC
SS5-R1	TCC ATT GAG TTG TCC ATT CC
GBSS-F1	GAC TGG AAA GAA GAA GAT GG
GBSS-R1	ACA TTA AGG GAC AAT AGT AC
18S rRNA-F	GGT AGG CGA TTG GCT AAC ATT GTC TGC
18S rRNA-R	GAG ACA CCA ACA GTC TTT CCT CTG CG
